# Cooperation mode selection and information sharing in a live streaming e-commerce supply chain with traffic data investment

**DOI:** 10.1371/journal.pone.0340203

**Published:** 2026-03-16

**Authors:** Huan Wang, Aimin Zhu, Lijuan Yu, Dai Mu, Zhiting Meng

**Affiliations:** 1 School of Management, Shenyang University of Technology, Shenyang, Liaoning, China; 2 Liaoning Academy of Social Sciences, Shenyang, Liaoning, China; Islamic Azad University Urmia Branch, IRAN, ISLAMIC REPUBLIC OF

## Abstract

In recent years, live streaming e-commerce has become an essential channel for brand merchants to expand online sales, with brand self-live streaming and influential streamer live streaming emerging as two mainstream cooperation modes. The choice between these modes directly affects brand merchants’ profit levels and the efficiency of supply chain coordination. However, the mechanisms through which platform information sharing and traffic data investment jointly influence brand merchants’ mode selection remain insufficiently explored. To address this gap, this paper constructs four game-theoretic models under scenarios of platform information sharing and non-sharing for both brand self-live streaming and influential streamer live streaming. The study systematically analyzes how factors such as demand information and traffic data investment affect the strategic decisions and profit structures of supply chain participants. The results show that: (1) the cost of traffic investment and the commission rate are key determinants of the brand merchant’s cooperation mode preference—when both are high, adopting the influential streamer live streaming mode yields greater benefits for the brand merchant; (2) the platform’s incentive to engage in information sharing varies significantly across cooperation modes, with a stronger motivation to share demand information under the brand self-live streaming mode; (3) and within a certain parameter range, the strategic interaction between information sharing and cooperation mode selection can foster a win–win outcome for both the brand merchant and the platform under the brand self-live streaming mode, thereby enhancing overall supply chain performance. This research elucidates the optimal cooperation mode selection logic of brand merchants in heterogeneous information environments and provides theoretical foundations and managerial insights for platform enterprises in designing effective information-sharing strategies and commission policies. To further verify the robustness and generalizability of the proposed models, several promising avenues for future research are also suggested.

## 1. Introduction

In recent years, the explosive growth of live streaming e-commerce has profoundly reshaped the traditional retail landscape. In 2023, the number of live streaming e-commerce users in China reached 530 million, accounting for 59.5% of total online shoppers, while the market size expanded to RMB 4.9 trillion, representing a year-on-year increase of 35.2% [1]. This innovative business model—characterized by real-time interactivity and scenario-based product presentation—has significantly enhanced product conversion rates, attracting numerous well-known brands such as L’Or éal and Gree to actively enter platforms like Taobao and Tik Tok to develop live streaming businesses. For example, Gree Electric Appliances, through live commerce hosted by “Dong Mingzhu,” achieved over RMB 10 billion in single-session sales during the “6·18” shopping festival, drawing wide public attention [2]. Similarly, L’Oréal leveraged Taobao live streaming to drive global performance, reaching total sales of EUR 38.261 billion in 2022 and achieving an operating profit growth of 21.06% [3]. These phenomena demonstrate that live streaming e-commerce has become a crucial pillar for brand digitalization and market expansion.

In practice, brand merchants primarily face two differentiated cooperation modes: brand self-live streaming and influential streamer live streaming. Under the brand self-live streaming mode, the brand merchant conducts live broadcasts via its own official account or brand channel, thereby maintaining full pricing autonomy, reinforcing brand image, and building consumer trust. However, this mode is essentially asset-intensive. For instance, Perfect Diary established its own live streaming studio to control both content and pricing, with self-live streaming contributing approximately 15%–20% of its total daily sales revenue. Although this approach effectively enhanced profitability, the brand encountered a growth bottleneck due to the high initial investment and limited audience coverage [4]. In contrast, influential streamer live streaming enables brand merchants to leverage the streamer’s fan base and platform recommendation algorithms to rapidly expand audience reach and generate short-term “blockbuster” effects. However, this mode requires the brand merchant to relinquish partial pricing power and profit margins. For example, Proya collaborated with well-known streamers and, through professional product presentations, significantly increased its audience exposure—its overall online search volume rose by about 400%, and profits improved markedly, albeit at the cost of diminished pricing control [4]. Hence, the core dilemma in brand merchants’ cooperation mode selection lies in the unique “traffic–demand” conversion mechanism of live streaming e-commerce, whereby traffic allocation directly determines market performance. Different modes entail systemic disparities in pricing authority, traffic investment incentives, and profit distribution mechanisms [5].

Against this backdrop, the driving role of traffic in shaping demand has become a key determinant of performance in live streaming e-commerce. Prior studies have emphasized that traffic operations are the core variable influencing sales conversion rates in live-streaming supply chains [6]. Structurally, total traffic in live streaming e-commerce can be classified into two categories: basic traffic and commercial traffic data. The former refers to the endogenous traffic owned by the streaming entity—either a brand merchant or an influential live streamer—and is jointly derived from private-domain followers and organic platform recommendations. It is characterized by being cost-free and stable [7]. In contrast, commercial traffic data refers to paid traffic resources purchased by brand merchants from platforms to increase short-term exposure and conversion rates [8]. For example, on the Tik Tok platform, the top two industries in terms of paid traffic purchases are personal care & cosmetics and apparel & accessories, whereas on Kwai, they are personal care & cosmetics and food & beverages [9]. Empirical evidence suggests that appropriate investment in commercial traffic data can significantly boost product sales and amplify brand exposure through synergistic interactions with the content innovation generated by influential live streamers. However, brand merchants’ decisions regarding commercial traffic investment are subject to high uncertainty. Since they are generally distant from the consumer end, it is difficult for them to acquire real-time market demand information, making it challenging to accurately predict the marginal returns of traffic investment. Under such circumstances, information sharing mechanisms have gradually emerged as a critical means for platforms and brand merchants to mitigate information asymmetry. For instance, JD.com collaborates with brands such as Midea and OPPO to share user behavior data and consumption trends, assisting them in optimizing traffic investment and pricing strategies [10]. While information sharing enhances supply–demand matching efficiency, it may simultaneously erode the platform’s bargaining power derived from information advantages, thereby triggering strategic interactions between the platform and brand merchants.

Therefore, exploring the optimal cooperation mode of brand merchants under the dual constraints of demand information sharing and commercial traffic investment has become a central issue in live streaming e-commerce supply chain management. To this end, this study constructs a multi-stage game model from the perspective of bilateral interaction between the brand merchant and the platform. Specifically, under the scenario where the platform may engage in demand information sharing, the model proceeds as follows: in the first stage, the brand merchant chooses the cooperation mode (brand self-live streaming or influential streamer live streaming) based on expectations about the platform’s information-sharing behavior; in the second stage, the platform decides whether to share demand information after observing demand signals; in the third stage, given the information-sharing status, both the brand merchant and the platform determine optimal pricing and traffic investment decisions to maximize their respective profits.

This framework systematically depicts the interaction mechanism between information sharing and traffic strategies, revealing their effects on brand merchants’ profits, platform performance, and overall supply chain efficiency. Based on the constructed multi-stage game model, the study provides an in-depth analysis of how different information-sharing strategies and traffic investment levels influence cooperation mode choice, platform revenue, and total supply chain performance. The findings offer theoretical foundations and practical implications for brand merchants in formulating optimal cooperation and traffic strategies, while also guiding platforms in optimizing information-sharing and traffic allocation policies. Overall, this research provides a novel analytical perspective for live streaming e-commerce operations, contributing to platform governance improvement and the sustainable development of digital commerce ecosystems.

The remainder of this paper is organized as follows: Section 2 provides a review of the relevant literature. Section 3 develops the model, Section 4 presents equilibrium analyses, Section 5 discusses equilibrium strategy combinations, Section 6 provides model extensions, and Section 7 concludes with key findings and managerial implications. All proofs are presented in the Appendix, which provides proofs of some thresholds, corollaries, lemmas, and propositions, as well as some extended equilibrium results.

## 2. Literature review

This study focuses on three key research areas in the e-commerce supply chain: traffic strategies, information sharing mechanisms, and cooperation mode selection. It systematically reviews and synthesizes relevant literature, traces theoretical developments and research evolution, and identifies the gaps and intersections between existing studies and this research.

### 2.1 Traffic strategies in e-commerce supply chains

Traffic strategies are widely recognized as critical determinants of sales performance in e-commerce. Existing research primarily explores this topic from three perspectives: upstream information transmission, midstream distribution efficiency, and downstream marketing conversion.

In terms of upstream information transmission, Zhou et al. [11] constructed a promotion budget and traffic allocation model based on historical data and machine learning. Their findings show that optimizing budget allocation can improve platform and merchant performance in the absence of pricing power. However, their model assumes a stable information environment and fails to account for how information asymmetry or selective disclosure by platforms feeds back into budget decisions. From the perspective of data power, Kirpalani and Philippon [12] argue that recommendation and matching technologies enhance efficiency but amplify platform market dominance, suggesting that traffic allocation carries governance and regulatory implications. Nevertheless, their model lacks a strategic representation of traffic as a purchasable short-term business domain resource. To overcome the limitations of static models, Shen et al. [13] proposed a multi-channel traffic allocation framework focused on differences in transaction volume and demand surges during promotional events. However, they did not embed traffic purchasing behavior within an asymmetric information game framework.

At the midstream stage, research has concentrated on the synergy among advertising bidding, recommendation algorithms, and forecasting methods. Chen et al. [14] integrated advertising auctions and organic recommendations within a dynamic optimization framework, showing that bidding strategies can amplify the benefits of recommendation traffic. Yet, this line of work emphasizes algorithmic optimization rather than merchants’ strategic decisions under information-sharing expectations. Lin et al. [15] enhanced traffic and sales forecasting accuracy using deep learning, providing empirical support for operations, though their prediction-oriented approach did not address how forecasted information is distributed and utilized in strategic games.

At the downstream level, Zhang et al. [16] empirically demonstrated that high-traffic key opinion leaders (KOLs) drive higher transaction volumes, whereas low-traffic KOLs improve investment returns, highlighting the heterogeneity of traffic effects. However, their conclusions, often based on specific product categories or platforms, cannot be easily generalized to theoretical frameworks involving information sharing and cooperation mode selection. Similarly, He et al. [17] examined short-video creation and traffic investment decisions in social e-commerce, showing that cost-sharing and value-allocation mechanisms can incentivize KOL participation. Nonetheless, their focus remains on cooperation mechanisms and does not incorporate platform demand information sharing into analysis.

### 2.2 Information sharing strategies in supply chains

Information sharing has long been viewed as a critical mechanism influencing supply chain coordination, profit distribution, and innovation incentives. Existing research can be broadly divided into two streams: one examines the game relationship between information sharing and sales mode decisions, and the other explores the interaction between information sharing and platform innovation or product development.

The first stream primarily employs game-theoretic models to analyze information-sharing incentives and outcomes between platforms and manufacturers or retailers [18]. For instance, Gong et al. [19] demonstrated that high commission rates and accurate information can jointly promote Pareto improvements in agency sales and information sharing. However, such studies mainly focus on traditional distribution frameworks, neglecting the marginal effects of traffic as a purchasable and strategic resource. Zhang and Nault [20] analyzed the impact of contractual structures on platform information-sharing motives, prices, and welfare, proposing price cap mechanisms to align interests. Yet, the applicability of such mechanisms to live streaming e-commerce remains uncertain due to the immediacy and short-term effects of paid traffic investment. Tian et al. [21] explored the complementary relationship between information sharing and agency sales in fresh produce e-commerce, providing category-specific insights but overlooking the strategic role of live streaming traffic investment.

The second stream emphasizes the role of information sharing in innovation and platform strategy. Niu et al. [22] found that sales data sharing in cross-border e-commerce enhances product development efficiency and achieves triple-win outcomes, highlighting its long-term innovation value, though they paid little attention to the short-term purchase of business domain traffic. Zhong et al. [23] revealed that platforms adopt differentiated sharing strategies based on competition structure and commission rates to manage encroachment and rivalry, but they did not incorporate the marginal returns of paid traffic into the core of sharing decisions.

Specialized studies on live streaming e-commerce remain in their infancy [24,25]. Lu et al. [26] examined how retailers’ information sharing affects manufacturers’ live-streaming channel expansion, revealing that the social influence of influential live streamers and channel expansion costs have non-monotonic effects on manufacturers’ profits. However, this work focuses on channel expansion rather than paid traffic investment by brand merchants. Ma and Liu [27] investigated how information disclosure costs and platform participation influence pricing and disclosure strategies, providing theoretical evidence of nonlinear effects of disclosure costs but without incorporating business-domain traffic as a key decision variable.

### 2.3 Cooperation mode selection in supply chains

Cooperation mode selection is a critical topic in platform and supply chain strategy research, yet it faces new institutional and technological challenges in the live streaming e-commerce context.

Traditional e-commerce studies on wholesale, reselling, agency, and hybrid modes have yielded extensive insights [28]. For example, Xiao et al. [29] found that platforms choose sales modes based on market size and corporate social responsibility (CSR) sensitivity; Yu et al. [30] analyzed strategic mode preferences between platforms and manufacturers; and Lin et al. [31] showed that multi-channel competition constrains strategic decisions. These studies provide robust methodological foundations for explaining conventional online retail behavior but overlook the impacts of live streamers, paid traffic acquisition, and platform information sharing.

Under information asymmetry [32], Chen et al. [33] demonstrated that information sharing and recommendation mechanisms influence platform choices between reselling and agency modes and that win–win outcomes can emerge under certain conditions. However, these models typically assume fixed traffic or demand signals, failing to endogenize brand merchants’ paid traffic investments. Tsunoda and Zennyo [34] further showed that platforms can adjust transparency and commission rates to influence suppliers’ cooperation modes, but their conclusions should be cautiously applied to live streaming e-commerce, where immediacy and traffic dominance are defining features.

Recent research has begun to specifically address mode selection in live streaming. Xin et al. [35] analyzed optimal product showcasing modes using Stackelberg games, identifying the effects of stimulation sensitivity and fixed participation costs. Yang et al. [36] compared multiple live-streaming sales modes, and Zhang et al. [37] examined the game between brand self-live streaming and influential streamer live streaming, suggesting that hybrid modes can outperform under certain conditions. These studies incorporate live-streaming characteristics into mode analysis for the first time, but they generally treat traffic as an exogenous condition or examine information disclosure only within a single mode. The interactive effects between demand information sharing and paid traffic investment remain insufficiently explored.

### 2.4 Summary

In summary, although existing studies have provided valuable insights into traffic strategies, information sharing mechanisms, and cooperation mode selection in e-commerce supply chains, several significant research gaps persist.

First, most studies treat business-domain traffic as an exogenous variable or technical parameter, neglecting its endogenous role in strategic games between brand merchants and platforms. This omission limits understanding of the feedback mechanisms between traffic investment and information sharing. Second, while the institutional implications of information asymmetry and platform governance have been widely acknowledged, systematic analyses of the joint decision-making processes involving information sharing and traffic investment are lacking—especially within the highly interactive and influencer-driven context of live streaming e-commerce.

To bridge these gaps, this paper develops a unified analytical framework that integrates platform information sharing strategies and business-domain traffic investment under conditions of information asymmetry and multi-stage decision-making in live streaming e-commerce. A Stackelberg game model is constructed to examine how platform information sharing influences brand merchants’ pricing and traffic allocation decisions under both brand self-live streaming and influential streamer live streaming modes, thereby revealing their optimal cooperation mode selection mechanism. Table 1 summarizes the comparison between this study and related literature.

**Table 1:** Comparison of relevant literature with our research.

Article	Supply chain type	Traffic data strategy	Information sharing strategy	Cooperation mode selection
Lin et al. [15]	Live streaming e-commerce	√		
He and Shang [17]	Social e-commerce	√		
Zhang et al. [16]	E-commerce supply chain		√	
Lu and Chen [25]	Live streaming e-commerce		√	
Tsunoda et al. [40]	E-commerce supply chain		√	√
Yang [28]	E-commerce supply chain			√
Yang et al. [36]	Live streaming e-commerce			√
Zhang et al. [37]	Live streaming e-commerce			√
This paper	Live streaming e-commerce	√	√	√

Based on this framework, the paper addresses three core research questions:

(1) How does platform information sharing affect brand merchants’ pricing and traffic investment decisions under different cooperation modes, thereby influencing mode selection?
(2) Does there exist an optimal information sharing strategy that enables the platform to maximize profit under varying commission rates and traffic costs?
(3) How does the interaction between information sharing and cooperation mode selection influence overall supply chain efficiency and profit distribution?

This study makes three major contributions. First, regarding brand merchant decisions, prior research has mainly focused on traditional e-commerce supply chains and analyzed how reselling and agency modes affect pricing and sales. Few studies have examined the joint influence of demand information sharing and traffic investment on cooperation mode decisions. This paper constructs a game-theoretic model incorporating these two factors, revealing how platform information sharing shapes brand merchants’ traffic allocation, pricing, and mode choices, thereby extending cooperation mode theory within live streaming e-commerce.

Second, from the platform’s perspective, the study identifies optimal information sharing strategies for profit maximization under varying commission rates and traffic costs. It further finds that when traffic investment costs are high, brand merchants’ cooperation mode choices can inversely influence the platform’s incentive to share information—indicating that even under high commission rates, brand merchants may prefer brand self-live streaming to gain an informational advantage. This extends the conclusions of Tsunoda and Zennyo [34], who found that brand merchants prefer agency sales under low commission rates.

Finally, regarding overall supply chain efficiency, the study demonstrates that the interaction between platform information sharing and brand merchants’ cooperation mode selection affects profit distribution and can achieve Pareto improvements—either through brand self-live streaming under high commission rates or influential streamer live streaming under low commission rates. This finding contrasts with Tan and Carrillo [38], who argued that agency selling is preferable under moderate commission rates in symmetric information settings, and extends Geng et al. [39] who suggested that reselling modes are always inferior. The results indicate that under the interplay between demand information sharing and cooperation mode selection, influential streamer live streaming may, in specific contexts, emerge as the optimal supply chain mode, providing a new theoretical basis for coordination between platforms and brand merchants.

## 3. Problem description and model assumptions

This section presents the problem under study, defines the notations used, and constructs the product demand function, basic assumptions, and information structure.

### 3.1 Problem description

**Fig 1:**
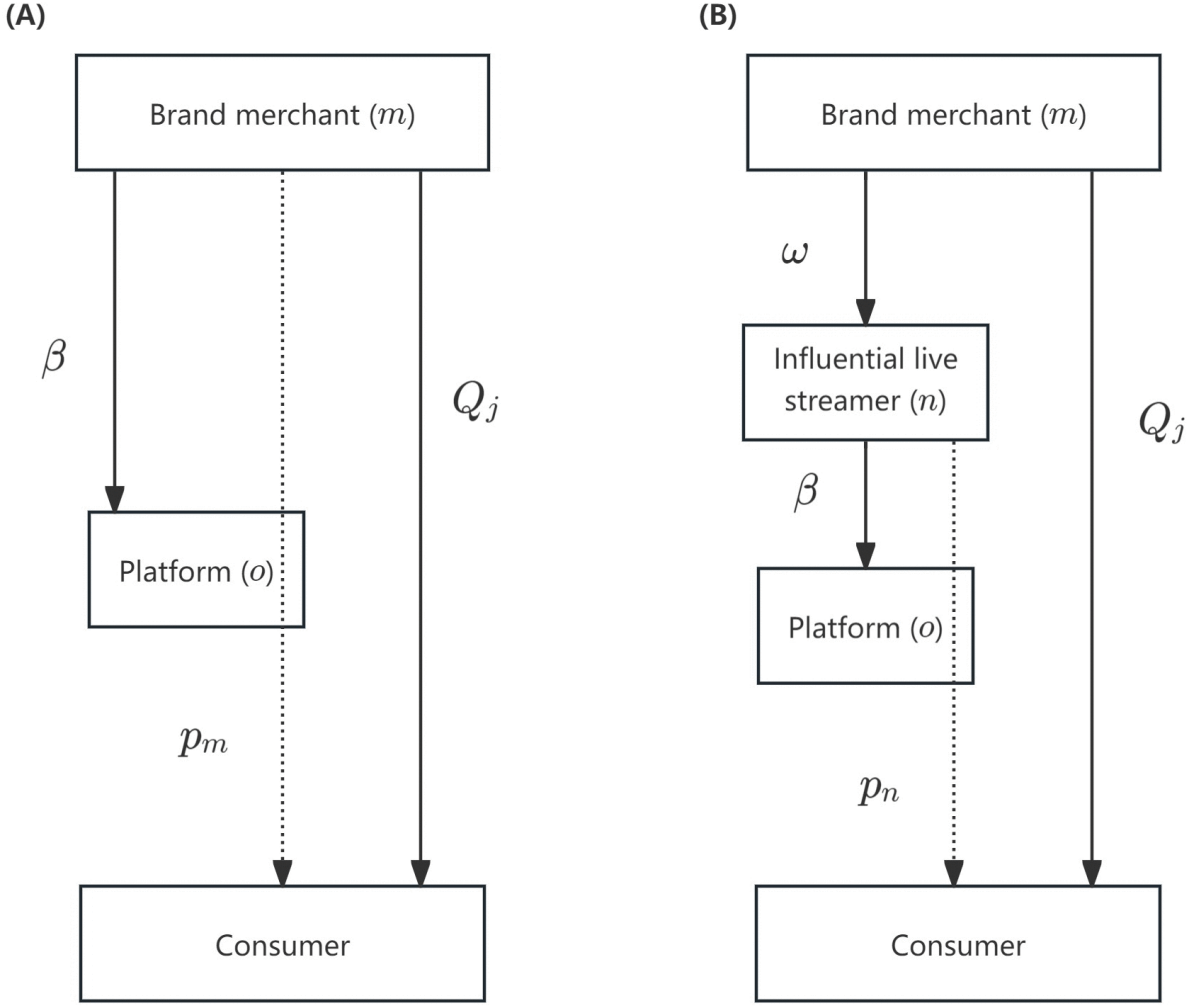
Supply chain structure. **(A)** Brand self-live streaming mode. **(B)** Influential streamer live streaming mode.

**Fig 2:**
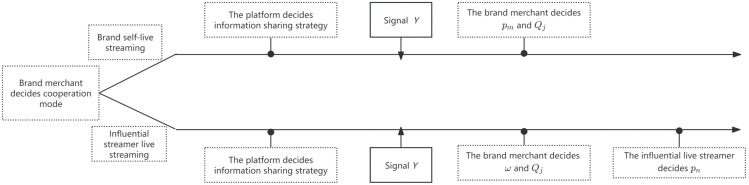
Sequence of decision.

Under demand information asymmetry, a live streaming e-commerce supply chain is composed of one brand merchant (denoted as *m*) and one live streaming platform (denoted as *o*). The brand merchant sells products to consumers through the live streaming platform, which offers two cooperation modes—brand self-live streaming and influential streamer live streaming. In the brand self-live streaming mode, the brand merchant conducts live streaming through its own account on the platform, selling products to consumers at a retail price $p_{m}\;$, while paying a commission at rate *β* to the platform, as illustrated in Fig 1(A). In contrast, in the influential streamer live streaming mode, the brand merchant sells products to an influential live streamer (denoted as *n*) at a wholesale price Ω. The streamer then sells the products at a retail price $p_{n}\;$ and pays the platform a commission at rate *β*, as shown in Fig 1(B). Additionally, the brand merchant invests in traffic acquisition by purchasing platform traffic ($Q_{j}\;$) to increase product exposure and stimulate market demand. The relevant notations are summarized in Table 2, and the decision sequence of the supply chain under different cooperation modes is shown in Fig 2.

**Table 2:** Definitions of variables and parameters.

Variables and parameters	Definition
Decision variable	
p	Product price.
$Q_{j}\;$	Level of traffic data investment in the commercial domain during live streaming ($0<Q_{j}<1\;$).
ω	Wholesale price of the products sold by the brand owner to the influential live streamer.
Other symbols	
i∈{m,n,o,mo,mno}	Supply chain members: *m* denotes the brand merchant, *n* the influential live streamer, *o* the live streaming platform; mo(mno represents the overall supply chain under the brand self-live streaming (influential streamer live streaming) mode.
X∈{A,R}	Cooperation mode: *A* denotes brand self-live streaming, *R* denotes influential streamer live streaming.
Z∈{I,N}	Information sharing strategy: *I* denotes information sharing, *N* denotes no information sharing.
a∈{H,L}	Market potential demand: *H* represents the high-demand state, *L* the low-demand state.
E[a|Y],E[π|Y],E[ω|Y]	Expected market potential demand, retail price, and wholesale price under information sharing.
E[a],E[π],E[ω]	Expected market potential demand, retail price, and wholesale price under no information sharing.
Y∈{h,l}	Market demand signal: *h* represents the high-demand signal, *l* the low-demand signal.
$\pi_{i}^{XZ}\;$	Profit of supply chain member *i* under strategy combination *XZ*.
d	Market demand.
r	Traffic-to-sale conversion rate during the live streaming (0<r<1 ).
β	Commission rate charged by live streaming e-commerce platform (0<β<1 ).
k	Cost coefficient of traffic investment in the commercial domain (0<k<1 ).
$Q_{j}^{s}\;$	Basic traffic (sum of private and public traffic) during live streaming, ($0<Q_{j}^{s}\;$).
ρ	Accuracy of market demand forecasting (1/2≤ρ≤1 ).
$V_{i}^{XZ}\;$	Incremental value of information sharing for member i under strategy combination *XZ*.

### 3.2 Basic assumptions

To construct the decision model for brand merchants under different live streaming modes, the following assumptions are proposed.

Assumption 1. Following the study of Qin et al. [8], this study assumes that the total traffic of the brand merchant consists of basic traffic ($Q_{j}^{s}\;$) and commercial-domain traffic ($Q_{j}\;$). The basic traffic refers to the sum of private-domain traffic derived from the intrinsic influence and follower base of the live streaming subject (brand merchant or influential live streamer) and the public-domain traffic generated by platform recommendations. This type of traffic is organic and cost-free. In contrast, commercial-domain traffic refers to the additional paid traffic that the brand merchant purchases from traffic service providers to rapidly expand exposure and reach a broader potential customer base within a short period. The information sharing behavior of the platform assists the brand merchant in determining optimal commercial-domain traffic investment, thereby mitigating market uncertainty.

Assumption 2. Following the study of Wang et al. [40], market demand is jointly influenced by the potential market size, total effective traffic, and traffic conversion rate. Accordingly, the market demand function is defined as


$$d=a-p+r(Q_{j}+Q_{j}^{s})\;$$ (1)

Here, *d* denotes the total market demand, *p* represents the product’s retail price, $Q_{j}^{s}\;$ refers to the basic traffic, and $Q_{j}\;$ denotes the commercial traffic data, jointly representing the total exposure traffic during live streaming. *r* represents the live streaming traffic conversion rate, which measures the impact of unit traffic on market demand. A higher *r* indicates a stronger ability of live streaming traffic to be converted into actual consumer demand. *a* denotes the market potential demand, which is subject to uncertainty and takes two states: high (a=H ) and low (a=L ). This binary state formulation is employed to characterize market uncertainty. We assume that the probability of the high and low states of potential market demand are equal, specifically Pr(a=H)=Pr(a=L)=1/2 . This probability is common knowledge among the live streaming platform, the influencer, and the brand merchant. Consequently, the expected (or average) market demand is given by $E[a]=\overset{―}{a}=(H+L)/2\;$. This base-case assumption is relaxed in Subsection 6.

Assumption 3. The traffic investment of the brand merchant incurs an operational cost. Following the studies of Cao et al. [41] and Wang et al. [9], the data-driven marketing cost is quadratically related to the investment. Specifically, the traffic investment cost is $1/2kQ_{j}^{2}\;$, where *k* is the coefficient for commercial traffic investment cost, and other operational costs are temporarily excluded from consideration.

Assumption 4. In accordance with Wang et al. [42] and Zhou et al. [43], live streaming e-commerce platforms typically charge a base commission rate of around 20%, though empirical evidence shows that major platforms (e.g., Taobao) often charge up to 30%. Considering that the platform’s adjustment of commission rates is a long-term strategic decision, the commission rate *β* is treated as an exogenous variable in this study. This setting aligns with the realistic assumption that platforms use commission rates as a long-term strategic tool rather than adjusting them in response to individual transactions. This base-case assumption is relaxed in Subsection 6.

Assumption 5. Following the studies of Liu et al. [44] and Zeng et al. [45], this study posits that top-tier influential live streamers (such as Li Jiaqi and Viya), who have accumulated extensive professional expertise, a large follower base, and deep insights into consumer online purchasing behaviors through long-term engagement in the live streaming e-commerce market, are capable of accurately perceiving the real state of potential market demand. Furthermore, to focus on the effects of different information structures and decision-making mechanisms, the theoretical model assumes identical traffic conversion rates and baseline traffic levels across the two cooperation modes, thereby enabling a clearer analysis of the game logic underlying information sharing and traffic investment decisions. In the theoretical model, abstract parameter settings—such as traffic cost, commission rate, and traffic conversion rate—are adopted to capture the essential characteristics of actual live streaming e-commerce operations. Although this abstraction simplifies market heterogeneity, it retains the core economic logic. Furthermore, parameter calibration and robustness verification based on realistic data are conducted in the subsequent numerical simulation section to ensure the validity of the analysis.

### 3.3 Information structure

With the development of big data and artificial intelligence, live streaming e-commerce platforms can accumulate large volumes of consumer data, enabling them to more accurately predict product demand. The platform obtains a demand forecast signal denoted as *Y*, where Y=h(Y=l represents a high (low) demand signal, corresponding to the underlying market demand state being *H* (high) or *L* (low). Following the study of Guo and Iyer [46], each signal Y∈h,l  is independently generated from the true state of demand a∈H,L  with probability ρ∈1/2,1 , where Pr(H|h)=Pr(L|l)=ρ . Similar information structures have been widely adopted in the literature [47–49]. Based on this framework, we derive Lemma 1.

**Lemma 1.** *(a) The probability of the live streaming platform observing* Y=h(Y=l *is given by*


$$Pr(h)=Pr(H)Pr(h|H)+Pr(L)Pr(h|L)=\frac{1}{2}\;$$

$$Pr(l)=Pr(H)Pr(l|H)+Pr(L)Pr(l|L)=\frac{1}{2}\;$$

*(b) The updated probability of the demand state conditional on signal* Y=h(Y=l *is*


Pr(H∣h)=Pr(L∣l)=ρ,Pr(H∣l)=Pr(L∣h)=1−ρ. 

*(c) The expected market potential demand conditional on signal* Y=h(Y=l *is*


$$\hat{H}=E[a|h]=\rho H+(1-\rho )L,\;\hat{L}=E[a|l]=\rho L+(1-\rho )H.\;$$

Here, *ρ* can be considered as the accuracy of market demand forecasting. For any given value of ρ∈[1/2,1] , there exists a condition where Pr(H|h)=Pr(L|l)≥Pr(H)=Pr(L holds true, indicating that as *ρ* increases, the demand-forecasting accuracy of the e-commerce platform improves.Furthermore, when ρ=1 , Pr(H|h)=Pr(L|l)=1  holds, which signifies a perfect forecasting signal for the e-commerce platform. Specifically, when the platform observes a high (low) forecasting signal, it corresponds to a high (low) potential market demand state. Conversely, when ρ=1/2  reaches a critical point, Pr(H|h)=Pr(H)=1/2  and Pr(L|l)=Pr(L)=1/2  holds, suggesting that the demand forecasting signal is ineffective at that moment.

## 4. Equilibrium analysis

Superscripts *X* and *Z* denote, respectively, the cooperation mode selected by the brand merchant and the information sharing strategy adopted by the live streaming e-commerce platform. Specifically, X=A(X=R indicates the brand self-live streaming (or influential streamer live streaming) mode, and Z=I(Z=N represents that the platform shares (or does not share) information. Accordingly, four strategic combinations are formed: (RI),(RN),(AI),(AN. Using the inverse order method, the optimal decisions and profits of both the brand merchant and the live streaming e-commerce platform are derived under each cooperation mode and information sharing strategy.

### 4.1 Brand self-live streaming(A)

When the brand merchant adopts the brand self-live streaming mode, if the live streaming e-commerce platform chooses to share information, the brand merchant can update its market demand expectation based on the observed signal *Y*. The expected profit function of the brand merchant is given by:


$$E[\pi_{m}|Y{]}^{AI}=(1-\beta )\;p_{m}\;E[d|Y]-\frac{1}{2}\;k\;Q_{j}^{2}\;$$ (2)

If the platform does not share information, the brand merchant can only rely on its own demand forecast, and its expected profit becomes:


$$E[\pi_{m}|Y{]}^{AN}=(1-\beta )\;p_{m}\;E[d]-\frac{1}{2}\;k\;Q_{j}^{2}\;$$ (3)

In the brand self-live streaming mode, the expected profit function of the live streaming e-commerce platform is expressed as follows:


$$E[\pi_{o}|Y{]}^{A}=\beta p_{m}E[d|Y$$ (4)

By applying the inverse order method, the optimal retail price and traffic investment level of the brand merchant, as well as the corresponding equilibrium profits of both the brand merchant and the platform, can be obtained as shown in Proposition 1.

**Proposition 1.** *In the brand self-live streaming mode, the optimal decisions and expected profits are:*


$$\begin{array}{c} p^{AI}=\frac{k(E[a|Y]+rQ_{j}^{s})}{2k+r^{2}(-1+\beta )},\;p^{AN}=\frac{k(\bar{a}+rQ_{j}^{s})}{2k+r^{2}(-1+\beta )},\\ Q_{j}^{AI}=-\frac{r(E[a|Y]+rQ_{j}^{s})}{2k+r^{2}(-1+\beta )},\;Q_{j}^{AN}=-\frac{r(\bar{a}+rQ_{j}^{s})}{2k+r^{2}(-1+\beta )},\\ E\pi_{m}^{AI}=\frac{k(E[a|Y]+rQ_{j}^{s}{)}^{2}\beta }{4k+2r^{2}(-1+\beta )},\;E\pi_{m}^{AN}=\frac{k(\bar{a}+rQ_{j}^{s}{)}^{2}(1-\beta )}{4k+2r^{2}(-1+\beta )},\\ E\pi_{o}^{AI}=\frac{k^{2}(E[a|Y]+rQ_{j}^{s}{)}^{2}\beta }{(2k+r^{2}(-1+\beta ){)}^{2}},\;E\pi_{o}^{AN}=\frac{k^{2}(\bar{a}+rQ_{j}^{s}{)}^{2}\beta }{(2k+r^{2}(-1+\beta ){)}^{2}}.\; \end{array}$$

As Proposition 1 indicates, changes in the traffic conversion rate will affect the product’s sales price and traffic investment level, and these effects are also influenced by variations in traffic costs. Therefore, the following analysis will examine the impacts of changes in commission rates, traffic cost coefficients, and traffic conversion rates, as detailed in Corollary 1.

**Corollary 1.** $(a\partial p^{A}/\partial \beta <0,\;\partial E\pi_{m}^{A}/\partial \beta <0,\;\partial Q_{j}^{A}/\partial \beta <0\;$. *As the platform’s commission rate* β  *increases, the brand merchant’s optimal price* $p^{A}\;$ *traffic investment* $Q_{j}^{A}\;$ *and profit* $E\pi_{m}^{A}\;$ *all decline.*

$(b\partial Q_{j}^{A}/\partial k<0,\;\partial p^{A}/\partial k<0\;$. *As the traffic cost coefficient k increases, the brand merchant’s optimal price and traffic investment decline.*

$(c\partial Q_{j}^{A}/\partial r>0\;$. *Traffic conversion efficiency improves with higher traffic investment.*

As stated in Proposition 1 and Corollary 1, these results indicate that, under the brand self-live streaming mode, the brand merchant’s product price, traffic investment, and profit all decrease with higher commission rates. The rationale is that a higher commission rate allows the platform to extract a larger share of revenue, thus compressing the merchant’s profit margin. To control costs, the brand merchant reduces traffic investment and lowers product prices to offset the demand loss caused by reduced exposure. When the commission rate is low, the merchant tends to increase traffic investment and raise prices moderately to enhance both brand visibility and profitability. Conversely, a higher traffic cost coefficient discourages traffic investment and pricing. As the marginal cost of traffic rises, the merchant strategically reduces investment to control expenditures, and the resulting demand decline compels further price reduction to sustain sales. Moreover, traffic investment tends to rise with higher traffic conversion rates since improved conversion enhances the marginal return of each traffic unit. Thus, merchants must balance traffic conversion efficiency and traffic cost when making investment decisions to maximize profit.

To further illustrate the joint effects of traffic conversion rate and traffic cost coefficient on traffic investment, a numerical example is provided in Table 3, following Wang et al. [40]. The parameters are set as $Q_{j}^{s}=50,a=200,and\;\beta =0.2\;$.

**Table 3:** Effects of traffic conversion rate and cost coefficient on traffic investment.

*r*	*k*			
	0.2	0.4	0.6	0.8
0.1	41.837	20.7071	13.7584	10.3015
0.2	91.3043	43.75	28.7671	21.4286
0.3	157.317	70.8791	45.7441	33.7696
0.4	258.824	104.762	65.6716	47.8261
0.5	450	150	90	64.2857

As shown in Table 3, the brand merchant’s traffic investment increases significantly with higher traffic conversion rates. This is because a higher conversion rate implies that each unit of traffic yields more sales conversions and stronger market demand, thus enhancing the marginal return on traffic investment. When the traffic cost coefficient is low, merchants improve conversion efficiency through enhanced content quality and interactivity while expanding traffic investment to boost sales scale. Conversely, under high traffic costs, merchants emphasize investment efficiency by improving organic traffic, brand appeal, and data-driven content strategies to alleviate cost pressure. This finding highlights that in live streaming e-commerce, improving the traffic conversion rate is essential for stimulating merchants’ willingness to invest in traffic and for achieving efficient utilization of traffic resources. From a managerial perspective, platforms should optimize recommendation algorithms, enhance content visibility, and provide data-driven tools to improve overall conversion efficiency. Furthermore, establishing a rational traffic pricing mechanism can guide brand merchants toward a “high conversion–high investment–high return” virtuous cycle, thereby promoting the sustainable development of the live streaming ecosystem.

### 4.2 Influencer-led live streaming model (R)

When the brand merchant adopts the influential streamer live streaming mode, and the live streaming e-commerce platform chooses to implement information sharing, the brand merchant can update its market demand forecast according to the demand signal *Y*. In this case, the expected profit function of the brand merchant is:


$$E[\pi_{m}|Y{]}^{RI}=\omega E[d|Y]-\frac{1}{2}kQ_{j}^{2}\;$$ (5)

The expected profit of the influential live streamer is:


$$E[\pi_{n}|Y{]}^{RI}=(1-\beta )(p_{n}-\omega )E[d|Y$$ (6)

If the live streaming e-commerce platform chooses not to share information, the brand merchant can only make decisions based on its own market demand expectations. The expected profit function of the brand merchant then becomes:


$$E[\pi_{m}|Y{]}^{RN}=\omega E[d]-\frac{1}{2}kQ_{j}^{2}\;$$ (7)

However, in Assumption 5, we propose that influential live streamers, having accumulated extensive experience in the live streaming e-commerce market, possess a deep understanding of consumers’ actual needs.At this point, the expected profit of the influencer is as follows


$$E[\pi_{n}|Y{]}^{RN}=(1-\beta )(p_{n}-\omega )E[d|Y$$ (8)

Under the influential streamer live streaming mode, the platform’s expected profit function is:


$$E[\pi_{o}|Y{]}^{R}=(1-\beta )(p_{n}-\omega )E[d|Y$$ (9)

According to the inverse order method, we obtain Proposition 2.

**Proposition 2.** *Under the influential streamer live streaming mode, the optimal decisions and expected profits of the brand merchant and the platform are as follows:*


$$\begin{array}{cc} p^{RI}=\frac{3k(E[a|Y]+rQ_{j}^{S})}{4k-r^{2}},\; & p^{RN}=\frac{2\bar{a}k+4kE[a|Y]+6krQ_{j}^{S}+\bar{a}r^{2}-4r^{2}E[a|Y]}{2(4k-r^{2})},\\ Q_{j}^{RI}=\frac{r(E[a|Y]+rQ_{j}^{S})}{-4k+r^{2}},\; & Q_{j}^{RN}=\frac{r(\bar{a}+rQ_{j}^{S})}{-4k+r^{2}},\\ \omega^{RI}=\frac{2k(E[a|Y]+rQ_{j}^{S})}{4k-r^{2}},\; & \omega^{RN}=\frac{2k(\bar{a}+rQ_{j}^{S})}{4k-r^{2}},\\ E\pi_{m}^{RI}=\frac{k(E[a|Y]+rQ_{j}^{S}{)}^{2}}{8k-2r^{2}},\; & E\pi_{m}^{RN}=\frac{k(\bar{a}+rQ_{j}^{S}{)}^{2}}{8k-2r^{2}},\\ E\pi_{n}^{RI}=\frac{k^{2}(E[a|Y]+rQ_{j}^{S}{)}^{2}(-1+\beta )}{(-4k+r^{2}{)}^{2}},\; & E\pi_{n}^{RN}=\frac{\left(-2(\bar{a}-2E[a|Y])k+2krQ_{j}^{S}+(\bar{a}-E[a|Y])r^{2}{)}^{2}(-1+\beta \right)}{4(-4k+r^{2}{)}^{2}},\\ E\pi_{o}^{RI}=\frac{k^{2}(E[a|Y]+rQ_{j}^{S}{)}^{2}\beta }{(-4k+r^{2}{)}^{2}},\; & E\pi_{o}^{RN}=\frac{(-2(\bar{a}-2E[a|Y])k+2krQ_{j}^{S}+(\bar{a}-E[a|Y])r^{2}{)}^{2}\beta }{4(-4k+r^{2}{)}^{2}}.\; \end{array}$$

From Proposition2, it is evident that under the influential streamer live streaming mode, the product resale price, retail price, and traffic investment level are jointly affected by the traffic cost coefficient and the traffic conversion rate, as summarized in Corollary 2.

**Corollary 2.** $(a\partial p^{R}/\partial k<0,\;\partial \omega^{R}/\partial k<0,\;\partial Q_{j}^{R}/\partial k<0\;$, *indicating that the commercial-domain traffic cost coefficient k has a negative effect on the product price, resale price, and traffic investment level.*

$(b\partial p^{R}/\partial r>0,\;\partial Q_{j}^{R}/\partial r>0,\;\partial \omega^{R}/\partial r>0\;$, *implying that a higher traffic conversion rate r positively influences product price, resale price, and traffic investment.*

Corollary 2 shows that in the influential streamer live streaming mode, the product price, resale price, and traffic investment level all decline with an increase in the traffic cost coefficient. The underlying mechanism is that as traffic costs rise, the marginal cost of obtaining traffic increases, prompting the brand merchant to reduce traffic investment and lower the resale price. In response, the influential live streamer may reduce the retail price to stimulate demand and maintain sales volume. Conversely, when the traffic conversion rate improves, the brand merchant can raise the resale price to capture higher profits, while the influencer is incentivized to enhance promotion efforts to increase sales. Under such conditions, the cooperation between the brand merchant and the influencer becomes more complementary, and both parties’ profits are positively correlated with the traffic conversion rate. A higher conversion rate strengthens consumer purchase intention, enabling influencers to modestly raise retail prices to expand their profit margins. Meanwhile, the brand merchant is more willing to increase traffic investment to expand realized demand. Therefore, the brand merchant should dynamically monitor conversion rate changes and intensify traffic investment when conversion efficiency is high to achieve dual optimization of market demand and profitability.

### 4.3 Sensitivity analysis

To examine the robustness of the model conclusions, this section conducts a sensitivity analysis on key parameters, namely the traffic cost coefficient, the platform commission rate, and the traffic conversion rate. These parameters directly influence the optimal decisions and profit levels of the brand merchant, the influential live streamer, and the platform. The results are summarized in Lemma 2.

To ensure analytical validity, parameter intervals are selected based on representative settings in the existing literature on live streaming e-commerce and supply chain pricing (e.g., Wang et al. [40]; Zhao et al. [50]. Moreover, to enhance empirical interpretability, the chosen parameter ranges draw on industry reports and publicly available traffic data. For the commission rate *β*, multiple industry reports indicate that commission rates in live streaming sales typically range between 10% and 30% [51–53]. For example, Douyin’s and Taobao’s commission rates are approximately 10% and 30%, respectively. Regarding the conversion rate *r*, empirical data show that average live streaming conversion rates generally range from 0.1% to 10%; typical live streams achieve around 2.1%, while premium streams such as “Oriental Selection” have reported conversion rates of 4.2% [54,55]. As for the traffic cost coefficient (k), paid traffic often accounts for 20–30% of total costs, while aggressive merchants may spend over 50% on traffic [56].

**Lemma 2.** *The effects of parameters k,β and r on the equilibrium results are shown in* Table 4.

**Table 4:** Sensitivity Analysis of Key Parameters and Equilibrium Outcomes.

Parameter	Mode	Ω	*p*	$Q_{j}\;$	$\pi_{m}\;$	$\pi_{o}\;$	$\pi_{n}$
k↑	AN	N/A	↓	↓	↓	↓	N/A
	AI	N/A	↓	↓	↓	↓	N/A
	RN	↓	↓	↓	↓	↓	↓
	RI	↓	↓	↓	↓	↓	↓
β↑	AN	N/A	↓	↓	↓	↑	N/A
	AI	N/A	↓	↓	↓	↑	N/A
	RN	–	–	–	–	↑	↓
	RI	–	–	–	–	↑	↓
r↑	AN	N/A	↑	↑	↑	↑	N/A
	AI	N/A	↑	↑	↑	↑	N/A
	RN	↑	↑	↑	↑	↑	↑
	RI	↑	↑	↑	↑	↑	↑

Notes: ↑: increasing; ↓: decreasing; –: no effect; N/A: Not relevant.

Lemma 2 indicates that variations in the key parameters significantly influence equilibrium outcomes and participants’ profits. First, as the traffic cost coefficient increases, product price, traffic investment, and profits for both the brand merchant and the platform decline, showing that high traffic costs weaken brand merchants’ incentives to invest in traffic, thereby suppressing market demand and overall supply chain performance. Second, an increase in the platform commission rate reduces the brand merchant’s profit and traffic investment under the brand self-live streaming mode, while boosting platform profit. This implies that the commission mechanism directly shapes merchants’ cooperation mode preference: higher commission rates motivate merchants to choose influential streamer live streaming to share platform costs, whereas lower rates encourage brand self-live streaming to maximize profits. Finally, higher traffic conversion rates simultaneously enhance prices, traffic investment, and profits for all parties, confirming that improving traffic quality and conversion efficiency can significantly strengthen supply chain performance. Comparing information sharing and non-sharing scenarios further reveals that information sharing can mitigate the negative effects of higher traffic costs and commission rates, while enhancing platform profits and supply chain coordination efficiency. In summary, the sensitivity analysis verifies the robustness of the equilibrium results. Although the model is theoretically abstract, it effectively captures real-world decision-making logic among brand merchants under different platform strategies. It provides managerial implications for platforms when designing information sharing policies and commission mechanisms: platforms should reasonably control traffic costs, optimize commission structures, and enhance traffic conversion efficiency to maintain ecosystem balance and foster collaborative value creation through information sharing.

## 5. Equilibrium strategy

Following the reverse-order solution approach, the subsequent analysis will first examine the information-sharing strategy of the live streaming e-commerce platform, followed by an evaluation of the brand’s choice of cooperation mode. Given the potential interactive effects between the selection of the cooperation mode and the platform’s information-sharing strategy, this section will also assess how these interactions influence both individual participants in the live streaming e-commerce supply chain and the overall supply chain performance.

### 5.1 Equilibrium information sharing strategy

First, we analyze the platform’s information sharing strategy under each given cooperation mode. To clearly represent the value created for each participant under information sharing, define $V_{i}^{RI}=E\pi_{i}^{RI}-E\pi_{i}^{RN}\;$ as the information sharing value under the influential streamer live streaming mode, and $V_{i}^{AI}=E\pi_{i}^{AI}-E\pi_{i}^{AN}\;$ under the brand self-live streaming mode. By comparing these values across supply chain members, we derive Proposition 3.

**Proposition 3.** *(a) Under the brand self-live streaming mode:* $V_{m}^{AI}>0,\;V_{o}^{AI}>0\text{ and }V_{mo}^{AI}>0\;$*,indicating that the platform will actively share demand information. Information sharing positively enhances brand merchant profits, platform revenue, and overall supply chain performance.*

*(b) Under the influential streamer live streaming mode: (i) If* k>1/2 , *then* $V_{o}^{RI}<0\;$*,and the platform will not share information. If* $r^{2}/4<k<r^{2}/2\;$*,then* $V_{o}^{RI}>0\;$, *and the platform will actively share information.*

*(ii)* $V_{m}^{RI}>0\;$, *meaning that information sharing is always beneficial to the brand merchant.*
*(iii) When* k>1/2  *holds,* $V_{n}^{RI}<0\;$, *indicating that information sharing is disadvantageous for the influential live streamer; however, when* $r^{2}/4<k<r^{2}/2\;$ *holds,* $V_{n}^{RI}>0\;$, *and information sharing benefits the influencer.*
*(iv) When* $k>k_{1}\;$ *holds,* $V_{mno}^{RI}<0\;$, *implying that information sharing reduces overall supply chain profits; When* $r^{2}/4<k<k_{1}\;$ *holds,* $V_{mno}^{RI}>0\;$, *and information sharing improves total supply chain performance. The specific expression for* $k_{1}\;$ *is provided in the Appendix.here.*

From Proposition 3 (a), under the brand self-live streaming mode, the platform always prefers to actively share demand information with the brand merchant. The rationale is that the platform’s main revenue source is commission. Information sharing allows the brand merchant to make more accurate pricing and traffic investment decisions, thereby stimulating demand and sales volume, which in turn increases the platform’s commission income. Thus, information sharing creates a win–win situation for both parties and improves overall supply chain efficiency.

From Proposition 3(b), under the influential streamer live streaming mode, the impact of information sharing on platform profits is uncertain, depending on both traffic investment and conversion efficiency. When traffic investment lies within a moderate range $r^{2}/4<k<r^{2}/2\;$, information sharing significantly improves conversion efficiency and sales, thereby increasing platform commissions and motivating information disclosure. However, when investment exceeds the threshold $k>r^{2}/2\;$, the rising cost of traffic investment reduces marginal returns, and even with shared information, the platform cannot offset the cost pressure—thus, its incentive to share declines.

For the brand merchant, information sharing consistently yields positive effects, as shared demand signals enable more accurate market forecasting and better optimization of pricing and traffic strategies. For the influential live streamer, the effect depends on the level of traffic cost: when costs are low $r^{2}/4<k<r^{2}/2\;$, efficient traffic investment creates favorable conditions for promotion, enhancing influencer sales and commission income. However, when costs are high $k>r^{2}/2\;$, reduced traffic investment diminishes conversion efficiency and influencer earnings, making information sharing potentially detrimental to them.

From a system perspective, the net benefit of information sharing depends on traffic cost levels. When costs are moderate $r^{2}/4<k<k_{1}\;$, information sharing fosters tri-party coordination among brand merchants, influencers, and platforms, improving pricing, marketing, and overall conversion efficiency. Yet, when costs exceed the critical value $k>k_{1}\;$ excessive investment leads to resource waste and coordination inefficiency, reducing total supply chain profit.

**Fig 3:**
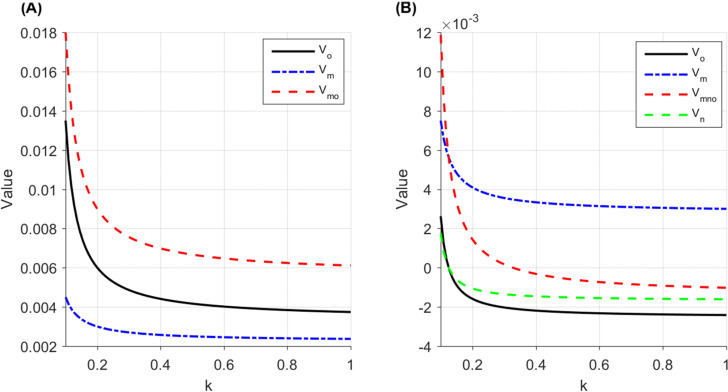
The value of information sharing under two cooperation modes. **(A)** Brand Self-live streaming mode **(B)** Influential streamer live streaming mode.

The following section further analyzes the value of information sharing among the members of the live streaming e-commerce supply chain within the regions discussed above, as illustrated in Fig 3. Specifically, Fig 3(A) and 3(B) represent the information-sharing value for the brand $(V_{m}$, the live streaming platform $(V_{o}$, the influential live streamer $(V_{n}$, and the supply chain $(V_{mo}^{AI},V_{mno}^{RI}$ under the brand self-live streaming mode and the influential streamer live streaming mode, respectively. Take H=1.5  L=1  ρ=0.8  r=0.5  β=0.6 , and verify the rationality of the model through drawing demonstration.

As shown in Fig 3(A), under the brand self-live streaming mode, information sharing always generates positive value for the brand merchant, platform, and the entire supply chain, though the marginal value declines as traffic cost increases. Fig 3(B) demonstrates that under the influential streamer live streaming mode, the value of information sharing similarly decreases with rising traffic cost. When traffic costs are relatively low $r^{2}/4<k<k_{1}\;$, information sharing benefits both the brand merchant and the overall supply chain; however, when $k>k_{1}\;$, its marginal benefit for the platform and influencer turns negative. This implies that at high traffic costs, the platform prefers information sharing under the brand self-live streaming mode but tends not to share information under the influential streamer live streaming mode, highlighting that the brand merchant’s cooperation mode choice significantly influences the platform’s information sharing strategy. Conversely, when traffic costs are low, the platform tends to share information under both modes, and the influence of cooperation mode on its information sharing decision becomes weaker.

**Fig 4:**
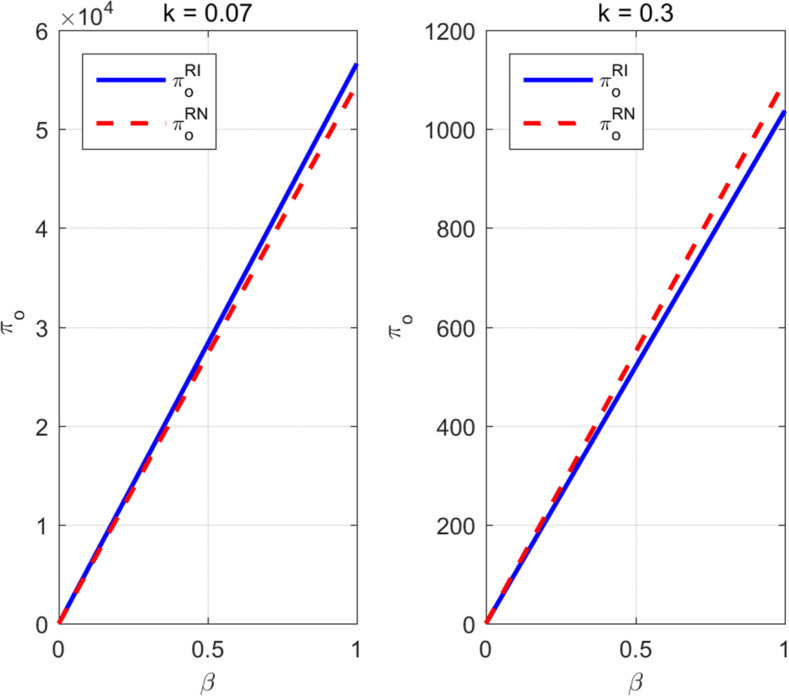
Strategic and parameter analysis of platform profit under influential streamer live streaming mode.

To further intuitively illustrate the impact of parameter value changes on platform profit under different information sharing strategies in the influential streamer live streaming mode, Fig 4 is plotted.

The two figures compare the trends in the live streaming e-commerce platform’s profit with respect to the commission rate under the influential streamer live streaming mode, at different traffic data investment costs (k=0.07  and k=0.3 ). The difference between the information sharing scenario (*RI*) and the non-sharing scenario (*RN*) is significantly moderated by the traffic data cost.At low traffic data cost (k=0.07 ), the platform profit in the RI mode is consistently and significantly higher than that in the RN mode. Both modes exhibit a linear increase as the commission rate rises, indicating that information sharing amplifies the positive effect of the commission rate on profit by optimizing efficiency. Conversely, at high traffic data cost (k=0.3 ), the profit curves for RI and RN nearly coincide. This suggests that the pressure from high traffic data investment costs diminishes the marginal benefit of information sharing, causing the difference in platform profit between the two cooperation modes to disappear, with profit being dominated only by the linear increase in the commission rate. Overall, the traffic data cost is the critical variable determining whether the advantage of the information sharing mode is significant: information sharing creates excess profit for the platform when traffic data cost is low, but its optimization effect is offset by cost pressure when traffic data cost is high, leading platform profit to primarily rely on the increase in the commission rate.

### 5.2 Equilibrium cooperation mode selection

In conjunction with the information sharing strategy of the live streaming platform, by comparing the brand merchant’s optimal profit levels under the brand self-live streaming mode and the influential streamer live streaming mode, the equilibrium cooperation mode of the brand merchant can be further derived. Thus, we obtain Proposition 4.

**Proposition 4.** *Equilibrium Cooperation Mode Selection of the Brand Merchant*

(a) *when* $k>\frac{r^{2}}{2}\;$ *and* $\beta_{1}<\beta <1\;$, *the equilibrium strategy combination of the brand merchant is the RN mode;*
(b) *when* $k>\frac{r^{2}}{2}\;$ and $0<\beta <\beta_{1}\;$, *or* $\frac{r^{2}}{4}<k<\frac{r^{2}}{2}\;$ *and* $\beta_{9}<\beta <\frac{1}{2}\;$, *the equilibrium strategy combination of the brand merchant is the AI mode;*
(c) *when* $k>\frac{r^{2}}{2}\;$ *and* $0<\beta <\beta_{9}\;$, *or* $\frac{1}{2}<\beta <1\;$, *the equilibrium strategy combination of the brand merchant is the RI mode.*

The specific expression of $\beta_{1}\;$ and $\beta_{9}\;$ are given in the Appendix.

According to Proposition 4, the brand merchant’s choice of cooperation mode is jointly influenced by the commission rate *β* and the traffic investment cost coefficient *k*. Different levels of commission and traffic cost not only affect the brand merchant’s profit structure but also alter the live streaming platform’s optimal decision on information sharing.

When the traffic investment cost is high, the platform tends not to share demand information in the influential streamer live streaming mode, while it is more inclined to share such information in the brand self-live streaming mode. Consequently, the brand merchant must simultaneously consider two factors when selecting a cooperation mode: (1) the platform’s information sharing strategy under each mode, and (2) the marginal effect of information sharing on its own profit. Since information sharing consistently enhances the brand merchant’s profit, it tends to prefer the cooperation mode that facilitates information sharing.

Specifically, when the commission rate is low, the brand merchant pays only a small proportion of commission to benefit from the platform’s information sharing, thus preferring the brand self-live streaming mode. However, when the commission rate is high, the brand merchant must balance the profit increase brought by information sharing and the cost pressure from higher commission payments. If the profit enhancement from information sharing is insufficient to offset the increased commission expenditure, the brand merchant is more likely to switch to the influential streamer live streaming mode to reduce total cost and improve marketing efficiency. Nonetheless, in certain high-commission ranges, brand merchants may still choose the self-live streaming mode because information sharing not only affects pricing and traffic decisions but also enhances investment–output efficiency through resource allocation optimization. When the platform’s demand forecasting accuracy is high, and market fluctuations are well captured, the additional profit from information sharing can offset the loss caused by high commission rates, maintaining the brand merchant’s incentive to adopt the self-live streaming mode.

When the traffic cost is low, the platform shares information in both the brand self-live streaming and influential streamer live streaming modes. In this case, the brand merchant’s cooperation mode selection no longer influences the platform’s information sharing strategy. The merchant’s choice depends more on the profit distribution structure and control of market power between the two modes.

Under such conditions, brand merchants exhibit distinct preferences across commission levels:

Low commission stage: Typically during the market introduction phase, merchants rely more on the private-domain traffic and fan stickiness of influential live streamers. The latter’s promotional capacity and reputation help rapidly build brand awareness and consumer trust, making the influential streamer live streaming mode more attractive.
Moderate commission stage: Merchants prefer the brand self-live streaming mode to maintain pricing autonomy and brand consistency while avoiding intermediary profit losses. This mode allows them to establish direct consumer relationships, improve customer experience, and foster repurchase behavior.
High commission stage: When commission costs approach the merchant’s upper tolerance limit, the influential streamer live streaming mode becomes an effective means to mitigate marketing risks. By incentivizing streamers as sales agents through resale or commission contracts, merchants can outsource marketing costs, reduce traffic investment risks, and improve return on investment.

Since the brand merchant’s cooperation mode choice, in turn, influences the platform’s incentive to share information, it is necessary to further analyze the feedback effect of the platform’s information sharing behavior under asymmetric information on the merchant’s mode selection. Accordingly, we propose the following Corollary 3:

**Corollary 3.** *(a) Under symmetric information, when* 0<β<1/2  *holds, the brand merchant selects the brand self-live streaming mode; when condition* 1/2<β<1  *holds, the brand merchant selects the influencer-led live streaming mode.*

*(b) In the case of asymmetric information, considering the e-commerce platform’s information-sharing strategy, the brand merchant selects influencer-led live streaming mode when condition* $0<\beta <\beta_{9}\;or\;1/2<\beta <1\;$ *holds, and selects brand self-live streaming mode when condition* $\beta_{9}<\beta <1/2\;$ *holds. The specific expression of* $\beta_{9}\;$ *is given in the Appendix.*

**Fig 5:**
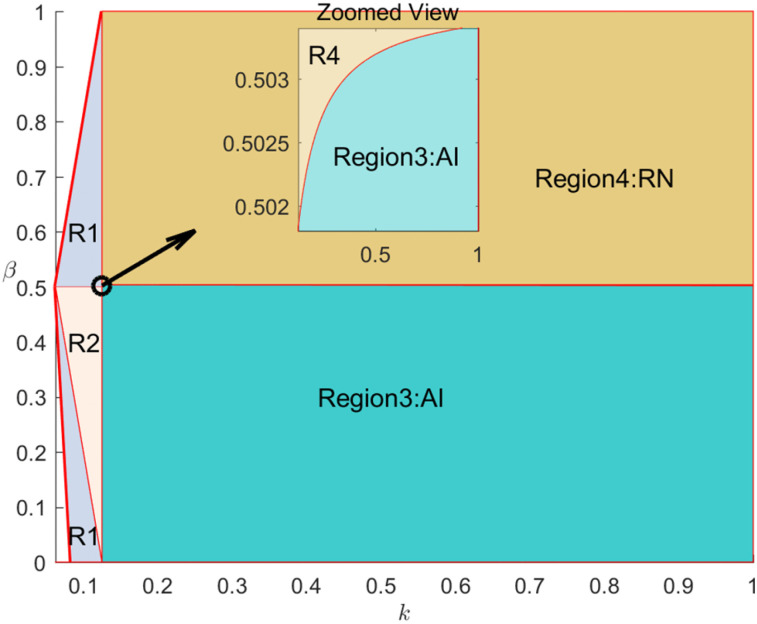
Brand’s equilibrium cooperation mode selection.

Fig 5 is presented to visually illustrate Proposition4 and Corollary 3.

Fig 5 presents the equilibrium regions of mode selection under different information structures. Under symmetric information, the merchant always selects the brand self-live streaming mode at low commission rates and the influential streamer live streaming mode at high commission rates. However, under asymmetric information where the platform decides whether to share demand information, we find that the merchant may still choose the brand self-live streaming mode even under higher commission rates. Further comparison indicates that, when considering the platform’s information sharing behavior under asymmetric information, merchants may shift from the influential streamer live streaming mode to the brand self-live streaming mode (e.g., region R2 in Fig 5). This is because, under asymmetric information, the platform tends to share information in the brand self-live streaming mode, and the additional profit from information sharing partially offsets commission costs, thereby expanding the region where merchants prefer self-live streaming. Hence, the platform’s information sharing strategy significantly affects the brand merchant’s cooperation mode selection. Practically, brand merchants can adjust their cooperation mode to promote information-sharing collaboration with platforms—for instance, for products with low traffic conversion rates (e.g., electronics or photographic equipment), merchants can adopt the self-live streaming mode to strengthen information-sharing cooperation with the platform.

### 5.3 The impact of strategic interactions on the live streaming e-commerce supply chain

There exists a significant interaction between the cooperation mode selection and the information sharing strategy, leading to differentiated impacts on the profits of supply chain members. This subsection investigates how such strategic interactions influence the brand merchant’s and platform’s payoffs, as well as the overall supply chain performance.

Following the study of Dan et al. [57], the brand merchant’s unilateral optimal strategy is defined as the cooperation mode that maximizes its expected profit without considering the platform’s strategy, while the platform’s unilateral optimal strategy refers to the information sharing choice that maximizes its own expected payoff regardless of the merchant’s mode. The feasible equilibrium set comprises all strategy combinations in which both parties obtain positive expected profits across the three cooperation modes (AI,RI,RN. Based on this, a Win–Win Outcome is defined as a specific Nash equilibrium in which both the platform and the merchant achieve their respective maximum expected profits within the feasible equilibrium set, reaching a Pareto-optimal outcome. Formally, when there exists a strategy combination *XZ* such that $E\pi_{o}^{XZ}=max\{E\pi_{o}^{AI},E\pi_{o}^{RI},E\pi_{o}^{RN}\}\;$ and $E\pi_{m}^{XZ}=max\{E\pi_{m}^{AI},E\pi_{m}^{RI},E\pi_{m}^{RN}\}\;$ the strategy combination *XZ* constitutes a Win–Win Outcome.

Conversely, a Lose–Lose Outcome arises from strategic misalignment, where the two sides’ unilateral optimal strategies diverge, resulting in a Pareto-suboptimal equilibrium. In this case, at least one party’s expected profit is lower than that obtainable in another feasible equilibrium, thereby weakening overall cooperative efficiency. Accordingly, we propose Proposition 5.

**Proposition 5.** *(a) When* $r^{2}/4<k<r^{2}/2,\;0<\beta <\beta_{10}\;or\;\beta_{11}<\beta <1\;$ *hold, the strategic interaction enables both parties to achieve their maximum payoffs under the RI equilibrium, indicating a Win–Win Outcome. When conditions* $r^{2}/4<k<r^{2}/2,\;\beta_{9}<\beta <\beta_{11}\;or\;k>r^{2}/2,\;0<\beta <\beta_{1}\;$ *are met, the interaction leads both sides to attain their highest profits under the AI equilibrium, also representing a Win–Win Outcome.*

*(b) When* $k>r^{2}/2,\;\beta_{1}<\beta <1\;$ *are met, both sides fail to achieve their optimal payoffs under the RN equilibrium, leading to a Lose–Lose Outcome.*

*The detailed expression of* $\beta_{1}\;$, $\beta_{9}\;$, $\beta_{10}\;$ *and* $\beta_{11}\;$ *are presented in the Appendix.*

**Fig 6:**
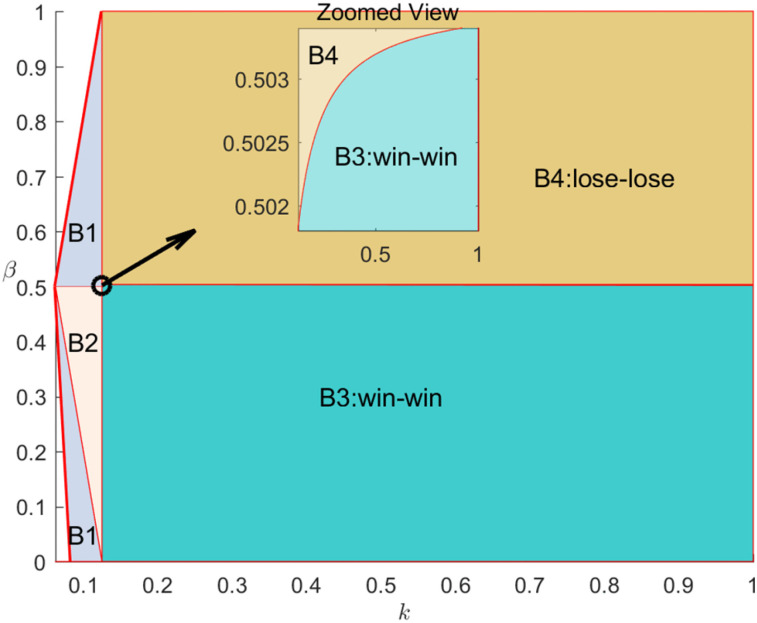
Impact of strategic interaction on live streaming e-commerce platform and brand.

Fig 6 illustrates these interaction effects.

Proposition 5 llustrates these interaction effects. Proposition 5 reveals that the interplay between information sharing and cooperation mode selection may generate either Win–Win or Lose–Lose outcomes. Generally, under low commission rates, brand merchants prefer the brand self-live streaming mode, whereas platforms tend to favor the influential streamer live streaming mode under high commission rates. However, Proposition 5(a) shows that under certain conditions (e.g., low commission and moderate traffic cost), both parties can align on the self-live streaming mode (AI strategy) to achieve a Win–Win outcome (regions B2 and B3 in Fig 6).

When the traffic cost coefficient is low, even if information is shared in the influential streamer live streaming mode, the resource improvement effect remains limited due to the low cost and commission levels. In contrast, in the self-live streaming mode, information sharing helps eliminate the double marginalization effect, leaving only the positive resource improvement effect. Therefore, within a moderate commission range, platforms prefer to implement information sharing under the self-live streaming mode. For traffic-insensitive durable goods (e.g., cameras, home appliances), such cooperation enhances the realization of a Win–Win outcome under the AI strategy.

Similarly, Proposition 5(a) indicates that under very low or very high commission rates (e.g., region B1 in Fig 6), the RI strategy can also achieve a Win–Win outcome. This is because in the moderate traffic cost range, the platform shares information under both modes, promoting coordinated pricing and traffic investment decisions. For traffic-sensitive high-frequency products (e.g., cosmetics, apparel, daily-use goods), cooperation under the RI strategy is more likely to achieve mutual benefit.

In contrast, Proposition 5(b) demonstrates that when both traffic cost and commission rate are high (e.g., region B4 in Fig 6), the RN strategy leads to a Lose–Lose situation. Here, strategy misalignment occurs: the brand merchant prefers the RN strategy to reduce dependency risk, while the platform favors AI to enhance data synergy. Such divergence weakens overall performance, reflecting the disruptive impact of strategic misalignment under asymmetric information.

Next, we will analyze the impact of the interaction between the choice of cooperation mode and information-sharing strategy on supply chain performance, as outlined in Proposition 6. For detailed resolution procedures, see section B of the Appendix.

**Proposition 6.** *The impact of strategic interaction on supply chain performance is as follows.*

(a) *When* $r^{2}/4<k<r^{2}/2,\;\beta_{2}<\beta <\beta_{9}\;or\;\beta_{9}<\beta <\beta_{3}\;$, *or when* $k_{1}<k<1,0<\beta <\beta_{3}\;$, *or when* $k_{1}<k<1,0<\beta <1\;$ *occur,* $E\pi_{mo}^{AI}>max\{E\pi_{mno}^{RI},E\pi_{mno}^{RN}\}\;$*. That is, the total supply chain profit under the AI strategy is superior to that under the RI and RN strategies.*
(b) *When* $r^{2}/4<k<r^{2}/2,0<\beta <\beta_{2}\;$, *or* $\beta_{3}<\beta <1\;$, *or when* $r^{2}/2<k<k_{1}\;$, $\beta_{3}<\beta <1\;$ *occur,* $E\pi_{mo}^{RI}>max\{E\pi_{mno}^{AI},E\pi_{mno}^{RN}\}\;$*. That is, the total supply chain profit under the RI strategy is superior to that under the AI and RN strategies.*

Proposition 6 indicates that the optimal strategy for supply chain performance depends on the interplay between the commission rate (*r*) and the traffic cost (*k*). When both the traffic cost and the commission rate are low, the RI strategy (influential streamer live streaming) enhances supply chain performance. In this scenario, the brand can rapidly establish market recognition through promotion by the influential live streamer, alleviating initial market entry pressure. This is particularly applicable to categories highly dependent on traffic conversion rates (e.g., daily necessities).

When the traffic cost is low and the commission rate is moderate, the AI strategy exhibits superior performance. Here, by leveraging the shared demand information in the brand self-live streaming mode, the brand can flexibly adjust its traffic investment, thereby achieving optimal resource utilization. The resource improvement effect, stemming from information sharing, enhances supply chain coordination efficiency. This is particularly suitable for medium-to-high-end durable goods where brand building and customer experience are emphasized. When the traffic cost is low and the commission rate is high, the RI strategy can still achieve a performance advantage. Under these conditions, the brand can effectively mitigate the double marginalization effect through influential streamer live streaming. It leverages the streamer’s influence to enhance product exposure and conversion, which is especially applicable to high-traffic, content-dependent products (e.g., cosmetics and apparel). In contrast, when the traffic cost is high, brands prefer the brand self-live streaming mode under a low commission rate to maintain autonomy over pricing and promotion and to control fixed costs. Conversely, under a high commission rate, the influential streamer live streaming mode is more cost-efficient, as the influential live streamer assumes a portion of the promotional risk and the pressure of traffic investment.

In general, the brand self-live streaming mode is analogous to a revenue-sharing contract, which facilitates supply chain coordination and overall performance optimization [58]. Meanwhile, the influential streamer live streaming mode, in the absence of information sharing, suffers from the double marginalization effect and is typically considered a suboptimal solution [39]. However, Proposition 6 highlights that with the introduction of an information sharing mechanism, the influential streamer live streaming mode can also enhance supply chain performance, which is attributed to the resource improvement effect derived from information sharing. In practice, mainstream live streaming e-commerce platforms have widely adopted such mechanisms. For example, Douyin provides data-empowered analysis for brands, Taobao Live generates big data reports based on product types, and JD Live supports brand decision-making through structured demand data, thereby significantly enhancing supply chain coordination efficiency and overall performance.

## 6. Extensions

To examine the robustness and general applicability of the core conclusions under more complex conditions, this section relaxes several fundamental assumptions and constructs four extended models for in-depth analysis. Specifically, these extensions include: incentive contract design, generalized demand probability, endogenous commission rate, and multi-brand competition. Drawing on the works of Zhang et al. [59], Duan et al. [60], Zhao et al. [61], Zhang and Ma [62], Dong et al. [63], and Peng et al. [64], we further explore additional scenarios incorporating factors such as multi-streamer competition, consumer heterogeneity, reputation dynamics, and platform competition. The analyses indicate that, although these extensions deepen the understanding of specific issues, they do not substantially alter the core managerial insights of this paper. Therefore, to maintain conciseness and focus in the main text, detailed derivations and analyses of these extensions are presented in Appendix.

### 6.1 Incentive contract design

Proposition 6 suggests that the most favorable cooperation strategies for the overall performance of the live streaming e-commerce supply chain are the RI strategy or the AI strategy, while the RN strategy is the least favorable. However, based on previous analyses, it is evident that in certain scenarios, the equilibrium strategy of the supply chain may be the RN strategy, which would clearly reduce supply chain performance. Therefore, the next step is to analyze whether there are scenarios within the live streaming e-commerce supply chain where performance improvements are achievable, and to design contracts tailored to these specific scenarios. By comparing the profits of e-commerce platforms, suppliers, and the overall supply chain under the three scenarios of RI, AI, and RN, Proposition 7 can be derived.

**Proposition 7.** *Identifies four scenarios in which a Pareto improvement in supply chain profits can be achieved, as shown in Table 5.*

**Table 5:** Scenarios leading to pareto improvements in supply chain performance.

Condition	Scenario 1	Scenario 2	Scenario 3	Scenario 4
	$k_{2}<k<1\;$	$\frac{r^{2}}{2}<k<k_{1}\;$	$\frac{r^{2}}{4}<k<\frac{r^{2}}{2}\;$	$\frac{r^{2}}{4}<k<\frac{r^{2}}{2}\;$
	$\beta_{1}<\beta <1\;$	$\beta_{7}<\beta <1\;$	$\beta_{9}<\beta <\frac{1}{2}\;$	$\beta_{2}<\beta <\beta_{9}\;$ or
				$\beta_{8}<\beta <\beta_{3}\;$
Equilibrium Strategy	RN	RN	AI	RI
Optimal Strategy for the Supply Chain	AI	RI	RI	AI
Platform Preferences	AI	RN	AI	AI
Brand Preferences	RN	RI	RI	RI
Transfer Payment Contracts	$F_{1}\;$	$F_{2}\;$	$F_{3}\;$	$F_{4}\;$

Note: The specific expressions of $k_{1},k_{2},\beta 1,\beta 2,\beta 3,\beta 7,\beta 8,\beta 9\;$ are provided in Appendix.

**Fig 7:**
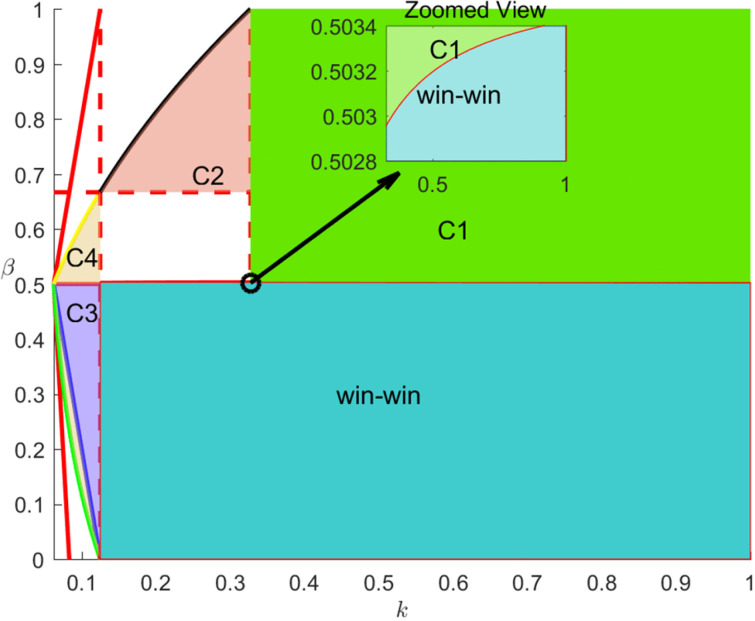
Supply chain performance pareto improvement.

To more clearly illustrate the various scenarios in Proposition 7, an analysis is conducted in conjunction with Fig 7.

Proposition 7 indicates that four distinct situations can achieve performance improvement in the live streaming e-commerce supply chain through appropriate transfer payment contracts:

Scenario 1: When traffic investment cost is high (Region C1 in Fig 7), the brand merchant’s optimal cooperation strategy is *RN*, while both the platform and the supply chain prefer *AI*. A subsidy $F_{1}\;$ from the platform to the brand merchant induces a shift from *RN* to *AI*, synchronously optimizing cooperation mode and information sharing.
Scenario 2: When traffic cost remains high (Region C2), the equilibrium strategy is *RN*, but the supply chain and brand merchant prefer *RI*. Here, a payment $F_{2}\;$ from the brand merchant to the platform incentivizes information sharing in the influential streamer live streaming mode, shifting cooperation from *RN* to *RI*, thus mitigating information asymmetry and sales risk.
Scenario 3: When traffic investment cost is low and the commission rate is high (Region C3), the platform prefers *AI* while the brand merchant and supply chain prefer *RI*. The brand merchant can pay $F_{3}\;$ to the platform to induce a shift from *AI* to *RI*, achieving risk-sharing and higher overall coordination.
Scenario 4: When both traffic cost and commission rate are low (Region C4), the brand merchant prefers *RI*, whereas the platform and supply chain prefer *AI*. A subsidy $F_{4}\;$ from the platform motivates the brand merchant to switch from *RI* to *AI*, thereby improving operational coordination and cooperation efficiency.

Overall, Proposition 7 highlights the critical role of fixed transfer payment contracts in promoting cooperation mode transformation and risk governance in live streaming e-commerce supply chains. The essence of such contracts lies in constructing incentive-compatible mechanisms, which align individual rational decisions with system-optimal outcomes through appropriate profit redistribution. The feasible range of each transfer payment $F\in (F_{\text{min}},F_{\text{max}}$ is determined by the relative bargaining power of the platform and the brand merchant. Proposition 8 summarizes these impacts.

**Proposition 8.** *The impact of the choice of cooperation mode and the adjustment of Information Sharing strategies on supply chain equilibrium decisions is shown in Table 6:*

**Table 6:** The impact of strategy adjustments on equilibrium decisions.

	Condition		Y=h	Y=l
Scenario 1(i)	$0<\beta <\beta_{4}\;$	RN→AI	$p^{*}↑,Q_{j}^{*}↑\;$	$p^{*}↓,Q_{j}^{*}↓\;$
Scenario 1(ii)	$\beta_{5}<\beta <1\;$	RN→AI	$p^{*}↓,Q_{j}^{*}↓\;$	$p^{*}↓,Q_{j}^{*}↓\;$
Scenario 2		RN→RI	$p^{*}↑,Q_{j}^{*}↑\;$	$p^{*}↓,Q_{j}^{*}↓\;$
Scenario 3(i)	$0<\beta <\beta_{6}\;$	AI→RI	$p^{*}↓,Q_{j}^{*}↓\;$	$p^{*}↓,Q_{j}^{*}↓\;$
Scenario 3(ii)	$\frac{1}{2}<\beta <1\;$	AI→RI	$p^{*}↑,Q_{j}^{*}↑\;$	$p^{*}↑,Q_{j}^{*}↑\;$
Scenario 4(i)	$0<\beta <\beta_{6}\;$	RI→AI	$p^{*}↑,Q_{j}^{*}↑\;$	$p^{*}↑,Q_{j}^{*}↑\;$
Scenario 4(ii)	$\frac{1}{2}<\beta <1\;$	RI→AI	$p^{*}↓,Q_{j}^{*}↓\;$	$p^{*}↓,Q_{j}^{*}↓\;$

Note:"→” indicates the shift in equilibrium strategy under the contract;"↑"and"↓"represent increases or decreases in equilibrium values;Y=h  and Y=l  denote high and low demand signals, respectively. The expressions for $\beta_{4},\beta_{5},\beta_{6}\;$ are provided in Appendix.

According to Proposition 8, in Scenario 1, when the brand merchant and the platform shift from the RN strategy to the AI strategy, the following patterns emerge: When the commission rate is relatively low and the market demand signal is strong, the brand merchant should increase both traffic investment and the selling price to enhance demand responsiveness and strengthen the brand premium effect. When the demand signal is weak, the brand merchant should reduce traffic investment and price to minimize costs and inventory risks. If the commission rate is high, regardless of the strength of the demand signal, the brand merchant should lower both traffic investment and price to mitigate commission pressure through a “quantity–price coordination” strategy. This implies that for products with low traffic conversion rates (such as electronic products or photographic equipment), a premium marketing strategy is appropriate under conditions of high demand and low commission, whereas a clearance pricing strategy is preferable under low-demand or high-commission conditions.

In Scenario 2, when the brand merchant and the platform shift from the RN strategy to the RI strategy, the brand merchant should increase traffic investment and price under high demand signals to capitalize on high marginal returns, and decrease both under low demand signals to avoid ineffective costs. This strategy is particularly suitable for products with high traffic conversion rates, such as cosmetics and fashion items, where a premium marketing strategy can be employed under high-demand conditions, while a discount promotion strategy is more appropriate under low demand.

In Scenario 3, when the brand merchant and the platform shift from the AI strategy to the RI strategy, if the commission rate is low, the brand merchant should reduce both traffic investment and price regardless of the demand signal. In this case, sales growth mainly relies on the fan base and promotional capability of the influential live streamer, where lower prices further enhance product competitiveness in streamer-led channels. If the commission rate is high, the brand merchant should moderately increase traffic investment and price to offset the cost pressure caused by high commissions. Therefore, for fast-moving consumer goods with high traffic conversion rates, a premium retention strategy is advisable under high-commission conditions, while a discount promotion strategy is preferable under low-commission settings.

In Scenario 4, when the brand merchant and the platform shift from the RI strategy to the AI strategy, under a low commission rate, the brand merchant should increase traffic investment and raise prices regardless of the market signal, leveraging the scale and brand premium effects associated with direct consumer engagement in brand self-live streaming. Under a high commission rate, however, both traffic investment and price should be reduced to stabilize profits through scale expansion and cost-sharing. Accordingly, for customized or durable products with relatively low traffic conversion rates (such as furniture and decorative items), a premium marketing strategy is more suitable in low-commission environments, whereas a clearance pricing strategy should be adopted under high-commission conditions.

### 6.2 Generalized demand probability setting

In the baseline model, the probabilities of high and low demand states were assumed equal, Pr(a=H)=Pr(a=L)=1/2 .This assumption, widely adopted in information sharing and game-theoretic studies, simplifies derivations and focuses analytical attention on the platform’s information sharing decision rather than on asymmetric prior probabilities. Statistically, in a two-state discrete demand system, equal probability corresponds to the case of maximum prior uncertainty (i.e., highest variance), representing a neutral baseline where the platform lacks prior market knowledge. However, in real markets, the probabilities of high and low demand are rarely symmetric; they may be influenced by policy guidance, marketing investment, and product life cycle. To enhance realism, following Iyer [49], we relax this assumption and let the probability of the high-demand state be *λ* (0<λ<1 ), and thus the low-demand probability 1−λ , We further investigate the impact of the demand structure on information sharing and cooperation mode selection strategies. To facilitate the analysis, we normalize the low demand state to 1 (i.e., L=1 ). Based on this, we obtain Lemma 3.

**Lemma 3.** *The probabilities that the platform observes a high-demand signal (h) and a low-demand signal (l) are given, respectively, by:*


Pr(h)=Pr(H)Pr(h|H)+Pr(L)Pr(h|L)=λ(2ρ−1)−ρ+1 

Pr(l)=Pr(L)Pr(l|L)+Pr(H)Pr(l|H)=λ(1−ρ)+(1−λ)ρ 

*Thus, the market demand state can be updated via Bayesian inference based on the demand forecast signal Y. The posterior probabilities are:*


$$Pr(H|h)=\frac{Pr(h|H)Pr(H)}{Pr(h)}=\frac{\lambda \rho }{(1-\lambda )(1-\rho )+\lambda \rho }\;$$

$$Pr(L|l)=\frac{Pr(l|L)Pr(L)}{Pr(l)}=\frac{(1-\lambda )\rho }{\lambda (1-\rho )+(1-\lambda )\rho }\;$$

$$Pr(H|l)=\frac{Pr(l|H)Pr(H)}{Pr(l)}=\frac{\lambda (1-\rho )}{\lambda (1-\rho )+(1-\lambda )\rho }\;$$

$$Pr(L|h)=\frac{Pr(h|L)Pr(L)}{Pr(h)}=\frac{\left(1-\lambda )(1-\rho \right)}{(1-\lambda )(1-\rho )+\lambda \rho }\;$$

*Conditional on observing the demand forecast signal Y, the updated potential market demand can be expressed as:*


$$\hat{H}=E[a|h]=HPr(H|h)+LPr(L|h)=\frac{\lambda (H\rho +\rho -1)-\rho +1}{\lambda (2\rho -1)-\rho +1}\;$$

$$\hat{L}=E[a|l]=HPr(H|l)+LPr(L|l)=\frac{\rho +\lambda (H-(1+H)\rho )}{\lambda +\rho -2\lambda \rho }\;$$

Accordingly, by applying Bayesian updating based on observed demand signals Y∈{h,l} , the posterior probabilities and expected market demands E[a|Y]  can be derived. Based on these results, define $V_{i}^{RI}=E\pi_{i}^{RI}-E\pi_{i}^{RN}\;$ as the information sharing value under the influential streamer live streaming mode, and $V_{i}^{AI}=E\pi_{i}^{AI}-E\pi_{i}^{AN}\;$ as that under brand self-live streaming. Combining these with Proposition 1, 2 and Lemma 3 yields the platform’s optimal information sharing strategy under generalized demand probability. Further comparing brand merchant profits across modes yields Proposition 9.

**Proposition 9.** *when* 1/2<β<1  *and* $r^{2}/4<k<r^{2}/2\;$, $E\pi_{m}^{RI}>E\pi_{m}^{AI}\;$ *and the equilibrium cooperation mode is RI; when* 0<β<1/2  *and* $(r^{2}-r^{2}\beta )/2<k<r^{2}/2\;$, $E\pi_{m}^{RI}<E\pi_{m}^{AI}\;$ *and the equilibrium cooperation mode is AI.*

This extension introduces asymmetry in demand probabilities and thereby enhances the model’s external validity. When the probability of a high-demand state increases, the market potential expands, and influential streamer live streaming (*RI*)becomes more profitable due to stronger market penetration and external traffic effects. Even with higher commission rates, the incremental marginal benefit outweighs the cost, making RI the preferred mode. Conversely, when the high-demand probability is low, demand uncertainty intensifies, and brand merchants favor brand self-live streaming (*AI*) to mitigate transaction risks and profit volatility. Under such conditions, direct control over pricing and traffic investment stabilizes earnings despite lower exposure. Hence, when 0<β<1/2  and $(r^{2}-r^{2}\beta )/2<k<r^{2}/2\;$, the *AI* mode remains optimal. This indicates that a higher likelihood of strong demand amplifies the leverage advantage of influential streamer live streaming, while a lower likelihood underscores the risk-hedging advantage of brand self-live streaming. Overall, introducing asymmetric demand probabilities enriches the theoretical model by revealing how brand merchants’ cooperation mode choices respond to demand expectations, traffic costs, and commission constraints, thus providing a more dynamic and realistic analytical framework.

### 6.3 Endogenous commission rate

In the baseline model, following the mainstream assumption adopted in most studies of live streaming e-commerce, the commission rate is treated as an exogenous parameter predetermined by the platform. This setting not only simplifies the analytical derivations but also highlights the mechanism through which the platform’s information sharing behavior influences the brand merchant’s cooperation mode selection. Moreover, it is consistent with the operational practice of most leading platforms, which typically adopt fixed commission rates. However, in certain market environments, platforms may dynamically adjust their commission rates in response to market performance or revenue-maximization objectives, thereby exhibiting endogenous characteristics. To enhance the model’s explanatory power and policy relevance, we further extend the baseline framework by treating the commission rate as an endogenous decision variable determined by the platform. This extension allows us to explore how a platform with pricing power strategically employs the commission mechanism to influence the brand merchant’s cooperation mode choice.

Following Zhou et al. [43], the platform determines the unit commission rate dynamically according to market conditions, and the live streamer subsequently pays the corresponding commission. Under this setting, the analysis in this section focuses on the influence of commission endogeneity on cooperation mode selection, while maintaining all other assumptions identical to those in the main model.

Accordingly, the expected profit functions of each supply chain member under the brand self-live streaming mode are expressed as follows:


$$\begin{array}{cc} E[\pi_{m}|Y{]}^{AI}=E[(p-\beta )d-\frac{1}{2}\;k\;Q_{j}^{2}|Y], & E[\pi_{o}|Y{]}^{AI}=E[\beta d|Y],\\ E[\pi_{m}{]}^{AN}=E[(p-\beta )d-\frac{1}{2}\;k\;Q_{j}^{2}], & E[\pi_{o}{]}^{AN}=E[\beta d].\\ \; \end{array}$$

Under the influential streamer live streaming mode, the corresponding expected profits are:


$$\begin{array}{cc} E[\pi_{m}|Y{]}^{RI}=E[(\omega d-\frac{1}{2}\;k\;Q_{j}^{2}|Y], & E[\pi_{o}|Y{]}^{R}=E[\beta d|Y],\\ E[\pi_{m}{]}^{RN}=E[(\omega d-\frac{1}{2}\;k\;Q_{j}^{2}], & E[\pi_{o}|Y{]}^{R}=E[(p-\omega -\beta d|Y].\\ \; \end{array}$$

By applying the inverse order method, the equilibrium solutions can be obtained. Combined with Lemma 1, the platform’s information sharing strategy is derived. Subsequently, comparing the brand merchant’s optimal profits under brand self-live streaming and influential streamer live streaming yields the equilibrium cooperation mode choice, leading to Proposition 10. The detailed proof is provided in the Appendix.

**Proposition 10.** when $r^{2}/2<k<1\;$, $E\pi_{m}^{RN}>E\pi_{m}^{AI}\;$, then the brand merchant’s equilibrium combination is the *RN* mode.

Compared with the baseline model, the introduction of endogenous commission rate alters the platform’s role from a passive participant to an active mechanism designer, thereby reshaping the brand merchant’s relative payoff structure. In the baseline model, where the commission rate is exogenously fixed, the platform lacks flexibility to adjust the revenue-sharing ratio based on market performance or traffic investment intensity. Under high traffic investment cost conditions $r^{2}/2<k<1\;$, the marginal return of brand self-live streaming (*AI*) diminishes, exposing the brand merchant to higher operational risk. In contrast, when the commission rate becomes endogenously determined, the platform optimizes the commission ratio according to market demand elasticity and the influencer’s selling capability. This dynamic adjustment enhances the influencer’s sales incentives. Consequently, by reselling products to the influencer, the brand merchant effectively outsources its sales and marketing functions to a more communication-efficient channel, thereby reducing both traffic investment costs and demand uncertainty risks. Meanwhile, the platform’s commission adjustment mechanism facilitates dynamic coordination among the brand merchant, the influencer, and the platform, aligning their incentives and improving overall supply chain efficiency. Thus, under endogenous commission conditions, the influential streamer live streaming (*RN*) mode becomes the brand merchant’s optimal strategy. This result underscores how internalized incentive mechanisms reshape the decision boundary and profit structure of brand merchants in live streaming e-commerce ecosystems.

### 6.4 Competition among brand merchants

While the baseline model focuses on a single brand merchant’s cooperation mode decision, this subsection extends the analysis to scenarios involving competition among brand merchants under information sharing. Specifically, consider a live streaming e-commerce platform (denoted by *o*) and an incumbent brand merchant ($m_{1}\;$) that adopts the brand self-live streaming mode to sell product $g_{1}\;$ through the platform. Merchant $m_{1}\;$ decides on the sales quantity $q_{1}\;$ and pays a commission rate *β* to the platform. Subsequently, a competing brand merchant ($m_{2}\;$) enters the platform to sell a substitutable homogeneous product $g_{2}\;$ Brand merchant $m_{2}\;$ invests in commercial-domain traffic $Q_{j}\;$ to increase product exposure and stimulate market demand. If $m_{2}\;$ adopts brand self-live streaming, it sells $g_{2}\;$ directly to consumers through the platform,decides its own sales quantity $q_{2}\;$, and pays a commission *β* to the platform. If $m_{2}\;$ adopts influential streamer live streaming, it resells product $g_{2}\;$ to an influential live streamer (*n*) at a wholesale price *omega*, who then sells $g_{2}\;$ to consumers via the platform, decides sales quantity $q_{2}\;$, and pays the same commission rate *β*.

Following Huang et al. [65], the inverse demand functions of the two products under information sharing are defined as: $p_{1}=a-q_{1}-bq_{2}\;$, $p_{2}=a-q_{2}-bq_{1}+rQ_{j}\;$, where b(0<b<1 ) denotes the intensity of market competition between the two brands—larger *b* values indicate fiercer competition. To isolate the mechanism of interest and exclude the influence of brand-specific traffic, we set the intrinsic brand traffic $Q_{j}=0\;$, as the analytical baseline. Additionally, we assume the commission rate satisfies 0<β<1/2 , a common condition in the literature [40], ensuring the existence of an interior optimal solution. All other assumptions and profit functions remain consistent with the baseline model.Using the inverse order method, we obtain the equilibrium outcomes and optimal profits of each supply chain member under competition, and then derive the optimal cooperation mode of the competing brand merchant. To ensure that the optimal solution under information sharing exists, we set the feasible profit region for the influencer live streaming mode of $m_{2}\;$ as: $0<b<b_{1}\;$, $r^{2}/4<k<k_{11}\;$, or $r^{2}-r^{2}\beta^{2}<k<k_{4}\;$, or $b_{1}<b<1\;$, $r^{2}/4<k<k_{3}\;$. Based on this, Proposition 11 is obtained. The proof is provided in the Appendix.

**Proposition 11.** *(a) If* $0<b<b_{1}\;$ *and* $\beta_{4}<\beta <1\;$ *with* $r^{2}-r^{2}\beta^{2}<k<k_{3}\;$,

*or* $0<r<r_{1}\;$, $0<b\leq b_{1}\;$, $0<\beta <\beta_{4}\;$ *with* $r^{2}-r^{2}\beta^{2}<k<k_{4}\;$,

*or* $\beta_{12}<\beta <1/2\;$ *and* $k_{3}<k<k4\;$,

*or* $r_{1}<r<1\;$, $0<b<b_{1}\;$,$0<\beta <\beta_{12}\;$ *with* $r^{2}-r^{2}\beta^{2}<k<k_{4}\;$, *then the brand merchant’s equilibrium strategy combination is the RI mode.*

*(b) If* $0<b<b_{1}\;$ *and* $k_{4}<k<k_{5}\;$,

*or* $0<b<b_{1}\;$ *and* $k_{5}<k<1\;$ *then the equilibrium strategy combination is the AI mode. The detailed expressions of* $k_{3},k_{4},k_{5},\beta_{12},b_{1},r_{1}\;$ *are given in the Appendix.*

In the extended model incorporating two competing brand merchants, brand self-live streaming with information sharing (*AI*) and influential streamer live streaming with information sharing (*RI*) emerge as the optimal cooperation modes due to the joint effects of information sharing and competitive interactions on traffic investment efficiency and profit allocation. From an economic perspective, the competition coefficient *b* introduces cross-effects in market demand: a brand merchant’s traffic investment influences not only its own sales but also imposes a crowding-out effect on competitors.When *b* is low (i.e., strong brand differentiation), information sharing effectively enhances the predictability of total market demand, reducing excessive traffic investment caused by uncertainty. In this case, the RI mode becomes optimal within certain parameter regions, as influential streamer live streaming—through higher traffic conversion efficiency and closer platform coordination—generates demand expansion benefits that outweigh commission costs. Conversely, when the traffic investment cost *k* is moderate to high, the commission rate is relatively low, and competition remains weak, the *AI* mode becomes optimal. Under information sharing, brand self-live streaming enables merchants to allocate traffic resources more precisely, control profit distribution, and reduce channel overlap losses stemming from competition.

Compared with the baseline model, the key difference lies in the redistribution of the value of information sharing. In a single-brand environment, information sharing mainly affects demand expectations and profit allocation. In a multi-brand competitive environment, however, information sharing simultaneously functions as a coordination mechanism that mitigates competitive uncertainty and enhances traffic investment efficiency. Therefore, under competition, both the *AI* and *RI* modes can emerge as equilibrium optima in different parameter regions, rather than the segmented optimal outcomes observed in the baseline model. This finding reveals that the interplay between information sharing and market competition redefines the strategic boundaries of cooperation mode choice in live streaming e-commerce supply chains.

## 7. Conclusion

### 7.1 Finding

With the rapid growth of live streaming e-commerce, platforms typically offer multiple cooperation modes to attract brand merchants. Consequently, brand merchants face the fundamental decision of selecting the optimal cooperation mode upon entering a platform. Since sales performance in live streaming relies heavily on traffic investment, limited knowledge of market demand may lead to inefficient investment and profit deviation. Under such conditions, the live streaming platform, which holds an information advantage regarding market demand, must decide whether to engage in information sharing with brand merchants. However, the profit distribution mechanisms differ significantly across cooperation modes, directly affecting the platform’s incentive to share information. Therefore, in deciding on a cooperation mode, brand merchants must consider not only cost and profit structures but also how their choice can facilitate information sharing and collaborative efficiency with the platform. Based on this rationale, this study constructs a bilateral supply chain model consisting of a brand merchant capable of traffic investment and a live streaming platform that possesses demand information superiority. The model systematically analyzes the brand merchant’s cooperation mode selection and the platform’s information sharing strategy.

The results show that the traffic cost coefficient and commission rate are key determinants of the brand merchant’s cooperation mode choice. Brand merchants can dynamically adjust their cooperation mode according to changes in these parameters to maximize overall performance. Further analysis reveals that shifts in cooperation mode significantly influence the platform’s information sharing strategy. Under the influential streamer live streaming mode, the platform’s willingness to share information is dynamically affected by traffic costs, whereas under the brand self-live streaming mode, the platform is more likely to engage in proactive and continuous information sharing. As a result, even under high commission rates, brand merchants may still prefer self-live streaming by adjusting traffic cost thresholds. Moreover, the interaction between traffic cost and commission rate can lead to a win–win equilibrium between the platform and the brand merchant under both AI (brand self-live streaming with information sharing) and RI (influential streamer live streaming with information sharing) modes. The findings also indicate that due to the strong interaction between cooperation mode and information sharing, the influential streamer live streaming mode can, within specific parameter ranges, yield the highest overall supply chain performance. This contrasts with prior studies that emphasized performance improvement under only one cooperation form—either agency or self-broadcasting. Finally, this study emphasizes that incentive contract design can help optimize strategic alignment between brand merchants and platforms, coordinating the relationship between information sharing and profit distribution. Under certain conditions, improving cooperation mode and information sharing mechanisms not only enhances supply chain coordination but also enables brand merchants to implement premium marketing strategies—achieving simultaneous improvements in brand value and market performance.

### 7.2 Managerial implications

The conclusions of this study provide important managerial insights into cooperation mode selection and information sharing strategies between brand merchants and platforms in live streaming e-commerce supply chains. First, both brand merchants and platforms should adopt differentiated mode selection based on traffic conversion characteristics. In practice, products with relatively low traffic conversion rates (e.g., electronics or customized furniture) require stronger trust building and product explanation. In such cases, the brand self-live streaming mode enables merchants to directly communicate brand value and product attributes to consumers. If platforms share traffic data on market demand, merchants can optimize live streaming content and traffic allocation schedules, improving conversion efficiency. Conversely, for products with higher traffic conversion rates (such as cosmetics and apparel), the influential streamer live streaming mode is more efficient in communication. Platforms should promote information sharing through transparent commission policies and data dashboards to foster collaborative marketing ecosystems. Real-world cases—such as Douyin’s influencer incentive program for beauty products and Alibaba’s strategic support for brand self-live streaming in home appliances—validate the effectiveness of combining differentiated modes with data-driven collaboration.

Second, supply chain participants should flexibly adjust pricing and traffic investment strategies according to product characteristics and market signals. For high-conversion products (e.g., fast-moving consumer goods), when the platform commission rate is high, brand merchants and influential live streamers may adopt a “premium preservation” strategy—using limited-time campaigns and refined content operations to enhance brand equity. Under low commission rates, a “discount promotion” strategy emphasizing volume growth and market penetration may be preferable. For low-conversion products (e.g., customized furniture or personalized decor), when the commission rate is high, a “clearance pricing” approach can help reduce inventory and increase exposure; when the commission rate is low, a “premium marketing” strategy can be used to reinforce brand identity and achieve profit maximization. Moreover, when market demand signals rise, merchants and streamers should adopt a “value-based pricing + brand exposure” strategy, whereas in a weak demand environment, they should focus on “promotion discounts + traffic stimulation” to balance short-term sales with long-term brand accumulation.

Third, attention should be given to how brand size and market demand heterogeneity affect optimal cooperation mode choice. Small brands, characterized by low recognition, weak fan bases, and high customer acquisition costs, are more dependent on platform traffic. During demand expansion, these brands should adopt the influential streamer live streaming mode to leverage streamers’ credibility and strong conversion ability for rapid brand exposure and user acquisition. Platforms, in turn, should provide demand forecasting data and traffic matching mechanisms to help small brands reduce decision uncertainty and improve live streaming efficiency. As the market matures or traffic costs rise, small brands can gradually transition to self-live streaming, focusing on content-based engagement to build private traffic and long-term brand assets. In contrast, large brands—equipped with stable audiences and strong content capabilities—benefit more from brand self-live streaming, which reinforces brand tone and value expression. Especially in high-demand contexts, information sharing under self-live streaming can further enhance traffic efficiency and profit margins. Therefore, when designing incentive and information sharing policies, platforms should consider brand size heterogeneity and use differentiated commission mechanisms and traffic allocation strategies to foster balanced ecosystem development. Overall, information sharing should be viewed not merely as a technological decision but as a strategic coordination mechanism. Through dynamic cooperation mode matching based on product attributes, brand scale, and market signals, both brand merchants and platforms can allocate resources more efficiently, optimize profit structures, and achieve sustainable growth within the diversified live streaming e-commerce ecosystem.

### 7.3 Research limitations and future directions

This study analyzes cooperation mode selection and profit optimization strategies in live streaming e-commerce using a game-theoretic framework, providing key insights into decision-making mechanisms between brands and platforms. However, several limitations remain. The analysis focuses on bilateral interactions between brands and platforms and does not explore qualitative factors such as brand image or influencer credibility, which are crucial in shaping consumer trust and engagement. Future research can incorporate emerging technologies such as artificial intelligence, blockchain, and big data analytics into the model to offer deeper insights into optimizing information sharing and traffic strategies within digital platforms.

## Supporting information

S1 Appendix The proof process of the relevant propositions and corollaries. (DOCX)
