# Supplementary material for: Cooperation mode selection and information sharing in a live streaming e-commerce supply chain with traffic data investment
Source: PLoS One. 2026 Mar 16;21(3):e0340203. doi: 10.1371/journal.pone.0340203 (PMC12991281; doi:10.1371/journal.pone.0340203)
Supplement: S1 Appendix — (DOCX) [file pone.0340203.s001.docx]

# **Cooperation Mode Selection and Information Sharing in a Live Streaming E-commerce Supply Chain With Traffic Data Investment Appendix**

The Appendix provides supplementary proofs and detailed analysis omitted from the main text, structured into four parts. Part A presents the mathematical expressions for the key thresholds. Part B contains the proofs for all propositions in the main text. Part C provides the supplementary analysis for the extended models, and Part D includes the proofs for the supplementary analysis of the extended models discussed in Part C.

# Part A. Mathematical expression of key thresholds

**Table A.1** Mathematical expressions of some critical thresholds

| Thresholds | Mathematical expressions |
| --- | --- |
| $k_{1}$ | $\frac{3r^{2}}{4}+\frac{1}{4}\sqrt{5}\sqrt{r^{4}}$ |
| $k_{2}$ | $\text{max}(\frac{r^{2}}{2},k_{1})$ |
| $k_{3}$ | $-\frac{r^{2}}{-8+b^{2}}$ |
| $k_{4}$ | $\frac{-48r^{2}+128r^{2}\beta+2b^{4}r^{2}\beta-64r^{2}\beta^{2}+16b^{2}r^{2}\beta^{2}-b^{4}r^{2}\beta^{2}}{8(-32+b^{4}+64\beta-16b^{2}\beta+b^{4}\beta)}+S_{1}$ |
| $k_{5}$ | $\frac{-r^{2}+r^{2}\beta}{-4+b^{2}}$ |
| $k_{6}$ | $\frac{12r^{2}-8b_{n}r^{2}+b_{n}^{2}r^{2}-8r^{2}\beta+4b_{n}r^{2}\beta}{16-6b_{n}^{2}+2b_{n}^{2}\beta}$ |
| $k_{7}$ | $\frac{-2r^{2}+2r^{2}\beta}{-8+4b_{o}^{2}+8\beta-4b_{o}^{2}\beta+b_{o}^{2}\beta^{2}}$ |
| $k_{8}$ | $\frac{-2r^{2}+2r^{2}\beta}{-4-4b_{o}+4b_{o}^{2}+4\beta+6b_{o}\beta-4b_{o}^{2}\beta-2b_{o}\beta^{2}+b_{o}^{2}\beta^{2}}$ |
| $\beta_{1}$ |  |
| $\beta_{2}$ | $\frac{2k}{8k-r^{2}}-\sqrt{2}\sqrt{\frac{16k^{3}-8k^{2}r^{2}+kr^{4}}{r^{2}(8k-r^{2})^{2}}}$ |
| $\beta_{3}$ | $\frac{2k}{8k-r^{2}}+\sqrt{2}\sqrt{\frac{16k^{3}-8k^{2}r^{2}+kr^{4}}{r^{2}(8k-r^{2})^{2}}}$ |
| $\beta_{4}$ | $1+\frac{2k(H+L+2Q_{j}^{s}r)(2k+r^{2})}{r^{2}(-2Hk-10kL-12kQ_{j}^{s}r-Hr^{2}+Lr^{2}+2(H-L)(-4k+r^{2})\rho)}$ |
| $\beta_{5}$ | $1-\frac{2k(H+L+2Q_{j}^{s}r)}{-(\text{（}H-L\text{）}r^{2}(-1+2\rho))+8k(L+Q_{j}^{s}r+H\rho-L\rho)}$ |
| $\beta_{6}$ | $\frac{2}{3}-\frac{2k}{3r^{2}}$ |
| $\beta_{7}$ | $\text{max}(\beta_{1},\beta_{3})$ |
| $\beta_{8}$ | $\text{max}(\frac{1}{2},\beta_{9})$ |
| $\beta_{9}$ | $\frac{-2k+r^{2}}{2}$ |
| $\beta_{10}$ | $\text{min}(\frac{-6k+2r^{2}}{r^{2}},\beta_{9})$ |
| $\beta_{11}$ | $\text{max}(\frac{1}{2},\frac{2k}{r^{2}})$ |
| $\beta_{12}$ | $\frac{-4+b^{2}}{-8+b^{2}}$ |
| $\beta_{13}$ | $\frac{-8+4b-b_{n}^{3}}{-16+2b_{n}^{2}}$ |
| $\beta_{14}$ | $\frac{-16k+6b_{n}^{2}k+12r^{2}-8b_{n}r^{2}+b_{n}^{2}r^{2}}{2b_{n}^{2}k+8r^{2}-4b_{n}r^{2}}$ |
| $b_{1}$ | $8\sqrt{2}\sqrt{\frac{88-20r^{2}+r^{4}}{(32-16r^{2}+r^{4})^{2}}}+\frac{8(-12+r^{2})}{32-16r^{2}+r^{4}}$ |
| $r_{1}$ | $\sqrt{\frac{2}{19+6\sqrt{10}}}$ |
| $\gamma_{m_{1}}$ | $\sqrt{\frac{\gamma_{n}^{2}-k\gamma_{n}^{2}-\beta\gamma_{n}^{2}}{(-1+2k)(-1+\beta)}}$ |
| $Q_{j_{1}}^{s}$ | $Q_{j_{1}}^{s}=\frac{1}{2}(\frac{(H+L)((-1-2k(-1+\beta)+\beta)\gamma_{m}+\gamma_{n}-(k+\beta)\gamma_{n})}{kr(-1+2\beta)}+L_{1}$ |

Note: $M_{1}=4(H-L)^{2}(4k-r^{2})\rho+4(H-L)^{2}(4k-r^{2})\rho^{2}$

，.

# Part B. Proofs of Propositions in the main paper

## Proof of Proposition 1

we verify that $E\pi_{s}^{A}$ is a concave function in terms of $p$ and $Q_{j}$, and derive its Hessian matrix as follows:

$$H(p,Q_{j})=\left(\begin{matrix} \frac{\partial^{2}E\pi_{m}}{\partial p^{2}} & \frac{\partial^{2}E\pi_{m}}{\partial p\partial Q_{j}} \\ \frac{\partial^{2}E\pi_{m}}{\partial Q_{j}\partial p} & \frac{\partial^{2}E\pi_{m}}{\partial p^{2}} \end{matrix}\right)=\left(\begin{matrix} -2+2\beta& r-r\beta\\ r-r\beta& -k \end{matrix}\right)$$

To ensure the Hessian matrix is negative definite, the parameters must satisfy the condition $-((2k+r^{2}(-1+\beta))(-1+\beta)>0$. Under this condition, let $\frac{\partial E\pi_{m}}{\partial p}=0$and $\frac{\partial E\pi_{m}}{\partial Q_{j}}=0$ be defined, allowing us to determine the equilibrium values of $p^{A*}$and $Q_{j}^{A*}$. In the case where the live streaming platform shares information, the brand can make decisions based on $E[a|Y]$. However, when the live streaming platform does not share information, the brand can only make decisions based on its own forecast of market demand, $\bar{a}$. This leads to the optimal decisions for the brand under the AI and AN strategies. By substituting the brand’s optimal decisions $p^{A*}$ and $Q_{j}^{A*}$ into the expected profit functions of the e-commerce platform and the brand, the expected profits can be derived for both parties.

## Proof of Corollary 1

From **Proposition 1**, we can easily get:$\hat{a}\in(E[a|Y]\text{，}\bar{a}$

,$\frac{\partial Q_{j}^{A*}}{\partial\beta}=\frac{(2k+r^{2})r(a+rQ_{j}^{s})}{(2k+r^{2}(-1+\beta))^{2}}<0$,$\frac{\partial E\pi_{m}^{A*}}{\partial\beta}=-\frac{4k^{2}(a+rQ_{j}^{s})^{2}}{(4k+2r^{2}(-1+\beta))^{2}}<0$.$\frac{\partial Q_{j}^{A*}}{\partial k}=\frac{2r(a+rQ_{j}^{s})(\beta-1)}{(2k+r^{2}(-1+\beta))^{2}}<0$,$\frac{\partial p^{A*}}{\partial k}=\frac{(a+rQ_{j}^{s})r^{2}(\beta-1)}{(2k+r^{2}(\beta-1))^{2}}<0$,$\frac{\partial Qj^{A*}}{\partial r}=\frac{2k(a+2rQ_{j}^{s})}{(2k+r^{2}(\beta-1))^{2}}>0$.

## Proof of Proposition 2

Proof: By applying backward induction, we first solve for the optimal decision of the influential live streamer. From equation $\frac{\partial^{2}E\pi_{\text{o}}}{\partial p^{2}}=-2\beta$, it follows that the profit function $p$ of the live streaming e-commerce platform is concave. Let $\frac{\partial E\pi_{n}}{\partial p}=0$ be defined, leading to $p^{R*}(\omega,Q_{j})=\frac{E[a|Y]+\omega+r(Q_{j}+Q_{s})}{2}$. If the e-commerce platform shares information, the brand makes decisions based on $p^{R*}(\omega,Q_{j})=\frac{E[a|Y]+\omega+r(Q_{j}+Q_{s})}{2}$; if the e-commerce platform does not share information, the brand makes decisions based on . Next, we verify the concavity of the brand’s profit function with respect to $\omega$ and $Q_{j}$. The Hessian matrix is derived as follows:

$$H(\omega,Q_{j})=\left(\begin{matrix} \frac{\partial^{2}E\pi_{m}}{\partial\omega^{2}} & \frac{\partial^{2}E\pi_{m}}{\partial\omega\partial Q_{j}} \\ \frac{\partial^{2}E\pi_{m}}{\partial Q_{j}\partial\omega} & \frac{\partial^{2}E\pi_{m}}{\partial Q_{j}^{2}} \end{matrix}\right)=\left(\begin{matrix} -1 & \frac{r}{2} \\ \frac{r}{2} & -k \end{matrix}\right)$$

To ensure the definiteness of the Hessian matrix, the parameters must satisfy condition $k\text{-}\frac{r^{2}}{4}>0$. Under this condition, let $\frac{\partial E\pi_{n}}{\partial p}=0$ and $\frac{\partial E\pi_{n}}{\partial p}=0$ be defined, which allows us to obtain the optimal values of $\omega^{R*}$and $Q_{j}^{R*}$. By substituting the expressions for $\omega^{R*}$ and $Q_{j}^{R*}$into $p^{R*}(\omega,Q_{j})$, the equilibrium value of$p^{R*}$is obtained. Finally, by substituting $\omega^{R*}$, $Q_{j}^{R*}$, and $p^{R*}$into the profit functions of the brand, the influential live streamer, and the live streaming platform, the optimal profits are derived.

## Proof of Corollary 2

It can be easily derived from **Proposition 2**.$\hat{a}\in(E[a|Y]\text{，}\bar{a}$

$\frac{\partial p^{R*}}{\partial k}=\frac{-3r^{2}(a+rQ_{j}^{s})}{(4k-r^{2})^{2}}<0$，$\frac{\partial\omega^{R*}}{\partial k}=\frac{-2r^{2}(a+rQ_{j}^{s})}{(4k-r^{2})^{2}}<0$；$\frac{\partial Q_{j}^{R*}}{\partial k}=\frac{-4r(a+rQ_{j}^{s})}{(-4k+r^{2})^{2}}<0$，$\frac{\partial p^{R*}}{\partial r}=\frac{3kQ_{j}^{s}(4k-r^{2})+6kr(a+rQ_{j}^{s})}{(4k-r^{2})^{2}}>0$，$\frac{\partial Q_{j}^{R*}}{\partial r}=\frac{(a+2rQ_{j}^{s})(4k-r^{2})+2ar^{2}+2Q_{j}^{s}r^{3}}{(-4k+r^{2})^{2}}>0$，$\frac{\partial\omega^{R*}}{\partial r}=\frac{2kQ_{j}^{s}(4k-r^{2})+4kr(a+rQ_{j}^{s})}{(4k-r^{2})^{2}}>0$.

## Proof of Proposition 3

According to **Proposition 1**, it follows that$V_{o}^{AI}=\frac{k^{2}(H-L)^{2}\beta(1-2\rho)^{2}}{4(2k+r^{2}(-1+\beta))^{2}}>0$，$V_{m}^{AI}=\frac{k(H-L)^{2}(-1+\beta)(1-2\rho)^{2}}{8(2k+r^{2}(-1+\beta))}>0$，$V_{mo}^{RI}=\frac{k(H-L)^{2}(2k-r^{2}(-1+\beta)^{2})(1-2\rho)^{2}}{8(2k+r^{2}(-1+\beta))^{2}}>0$，$V_{mo}^{AI}=V_{m}^{AI}+V_{o}^{AI}>0$；

By **Proposition 2**,$V_{o}^{RI}=-\frac{(H-L)^{2}(12k^{2}-8kr^{2}+r^{4})\beta(1-2\rho)^{2}}{16(-4k+r^{2})^{2}}$ it can be easily derived that: if $\frac{r^{2}}{4}<k<\frac{r^{2}}{2}$, then $V_{o}^{RI}>0$; if $k>\frac{r^{2}}{2}$, then $V_{o}^{RI}<0$. Similarly, it can be concluded that $V_{m}^{RI}=E_{m}^{RI}-E_{m}^{RN}=\frac{k(H-L)^{2}(1-2\rho)^{2}}{32k-8r^{2}}>0$.

From $V_{n}^{RI}=-\frac{(H-L)^{2}(12k^{2}-8kr^{2}+r^{4})(-1+\beta)(1-2\rho)^{2}}{16(-4k+r^{2})^{2}}$, it follows that when $\frac{r^{2}}{4}<k<\frac{r^{2}}{2}$, $V_{n}^{RI}>0$. When $k>\frac{r^{2}}{2}$, $V_{n}^{RI}<0$.

From $V_{mo}^{RI}=V_{m}^{RI}+V_{n}^{RI}+V_{o}^{RI}=-\frac{(H-L)^{2}(4k^{2}-6kr^{2}+r^{4})(1-2\rho)^{2}}{16(-4k+r^{2})^{2}}$, it follows that when $\frac{r^{2}}{4}<k<\frac{3r^{2}}{4}+\frac{1}{4}\sqrt{5}\sqrt{r^{4}}$,$V_{mno}^{RI}>0$. If $k>\frac{3r^{2}}{4}+\frac{1}{4}\sqrt{5}\sqrt{r^{4}}$, then $V_{mno}^{RI}<0$.

## Proof of Proposition 4

Case 1: When $k>\frac{r^{2}}{2}$, it follows that

$\begin{matrix} \Delta E\pi_{m}=E\pi_{m}^{AI}-E\pi_{m}^{RN} \\ =-\frac{k(\frac{1}{2}(\beta-1)(4k-r^{2})(\rho H-(1-\rho)L+rQ_{j}^{s})^{2}+(-(1-\rho)H+\rho L+rQ_{j}^{s})^{2})+(2k+r^{2}(\beta-1))(\frac{1}{2}(H+L)+rQ_{j}^{s}))^{2}}{2(2k+r^{2}(\beta-1))(4k-r^{2})} \end{matrix}$Subsequently, when $\beta_{1}<\beta<1$, $E\pi_{m}^{AI}-E\pi_{m}^{RN}=\Delta E\pi_{m}^{AI-RN}<0$, and when $0<\beta<\beta_{1}$, $E\pi_{m}^{AI}-E\pi_{m}^{RN}=\Delta E\pi_{m}^{AI-RN}>0$.

Case 2: When $\frac{r^{2}}{4}<k<\frac{r^{2}}{2}$, it follows that

$\begin{matrix} \Delta E\pi_{m}^{AI-RI}=E\pi_{m}^{AI}-E\pi_{m}^{RI} \\ =-\frac{k^{2}(-1+2\beta)(H^{2}+L^{2}-2HQ_{j}^{s}r+2LQ_{j}^{s}r+2Q_{j}^{s^{2}}r^{2}-2(H-L)^{2}\rho+2(H-L)^{2}\rho^{2})}{(16k-4r^{2})(4k+2r^{2}(-1+\beta))} \end{matrix}$Subsequently, when $\frac{-2k+r^{2}}{r^{2}}<\beta<1$, $E\pi_{m}^{AI}-E\pi_{m}^{RN}=\Delta E\pi_{m}^{AI-RN}>0$, and when $0<\beta<\frac{-2k+r^{2}}{r^{2}}or\frac{1}{2}<\beta<1$, $E\pi_{m}^{AI}-E\pi_{m}^{RN}=\Delta E\pi_{m}^{AI-RN}<0$.

## Proof: of Corollary 3

Under symmetric information, it can be easily derived that the profit of the brand merchant in the influential streamer live streaming mode is , while the profit in the brand self-live streaming mode is . Thus, it follows that$E\pi_{m}^{R*}-E\pi_{m}^{A*}=-\frac{k(H+L+2rQ_{j}^{s})^{2}(2k+r^{2}(\beta-1)+(\beta-1)(4k-r^{2}))}{4(2k+r^{2}(\beta-1))(4k-r^{2})}$holds. When condition $\frac{1}{2}<\beta<1$ holds, the brand merchant chooses the brand self-live streaming mode under symmetric information; when condition $0<\beta<\frac{1}{2}$ holds, the brand merchant opts for the influential streamer live streaming mode. Finally, combining **Proposition 4** leads to the conclusion of **Proposition 3.**

## Proof of Proposition 5

$M_{1}=4(H-L)^{2}(4k-r^{2})\rho+4(H-L)^{2}(4k-r^{2})\rho^{2}$ holds, we prove that

$$E\pi_{o}^{RN*}-E\pi_{o}^{AI*}=\frac{1}{8}\beta\left(-\frac{f(\rho)_{1}}{2k+r^{2}(-1+\beta)^{2}}+\frac{f(\rho)_{2}}{4(-4k+r^{2})^{2}}\right)$$

$$f(\rho)_{1}=4k^{2}(L+Q_{j}^{s}r+H\rho-L\rho)^{2}-4k^{2}(H+Q_{j}^{s}r-H\rho+L\rho)^{2}$$

$\begin{matrix} f(\rho)_{2}=4kQ_{j}^{s}r+kL(6-8\rho)+Hr^{2}(1-2\rho)+Lr^{2}(1-2\rho)+Hk(-2+8\rho)^{2} \\ +4kQ_{j}^{s}r+kH(6-8\rho)+Lr^{2}(1-2\rho)+Hr^{2}(1-2\rho)+Lk(-2+8\rho)^{2} \end{matrix}$，，$E\pi_{o}^{RI}-E\pi_{o}^{RN}=V_{o}^{RI}$. By comparing the symbols of $E\pi_{o}^{RN}-E\pi_{o}^{AI}$，$E\pi_{o}^{RI}-E\pi_{o}^{AI}$ and $V_{o}^{RI}$, it is evident that when$\frac{r^{2}}{4}<k<\frac{r^{2}}{2}$，$\frac{-2k+r^{2}}{r^{2}}<\beta<\text{min}\left(\frac{1}{2},\frac{2k}{r^{2}}\right)$ hold, or when $k>\frac{r^{2}}{2}$，$0<\beta<\beta_{1}$are true, $E\pi_{o}^{AI}>\max(E\pi_{o}^{RI},E\pi_{o}^{RN})$ results; and when $\frac{r^{2}}{4}<k<\frac{r^{2}}{2}$，$0<\beta<\text{min}\left(\frac{-6k+2r^{2}}{r^{2}},\frac{-2k+r^{2}}{r^{2}}\right)$ or $\text{max}\left(\frac{1}{2},\frac{2k}{r^{2}}\right)<\beta<1$ hold, $E\pi_{o}^{RI}>\max(E\pi_{o}^{AI},E\pi_{o}^{RN})$ follows.

Finally, by integrating **Proposition 4**, **Proposition 5** is derived.

## Proof of Proposition 6

$$\Delta E\pi_{mo}^{RI-AI}=E\pi_{mno}^{RI}-E\pi_{mo}^{AI}$$

$=-\frac{k^{2}(4k^{2}+r^{4}\beta-2kr^{2}(1-2\beta+4\beta^{2}))(H^{2}+L^{2}+2HQ_{j}^{s}r+2LQ_{j}^{s}r+2Q_{j}^{s}r^{2}-2(H-L)^{2}\rho+2(H-L)^{2}\rho^{2})}{2(-4k+r^{2})^{2}(2k+r^{2}(-1+\beta))^{2}}$ Thus, it follows that when$\frac{r^{2}}{4}<k<\frac{r^{2}}{2}$，$0<\beta<\beta_{2}$ or $\beta_{3}<\beta<1$ occurs, or when $k_{1}<k<1$,$0<\beta<1$ occurs, the condition $E\pi_{mo}^{AI}>\max(E\pi_{mno}^{RI},E\pi_{mo}^{RN})$ holds; when $\frac{r^{2}}{4}<k<\frac{r^{2}}{2}$, $0<\beta<\beta_{2}$, or $\beta_{3}<\beta<1$ occurs, or when $\frac{r^{2}}{2}<k<k_{1}$,$\beta_{3}<\beta<1$ occurs, the condition $E\pi_{mno}^{RI}>\max(E\pi_{mo}^{AI},E\pi_{mno}^{RN})$holds.

## Proof of Proposition 7

Proof: Integrating **Proposition 4**, **Proposition 5**, and the intervals for comparing brand profits, platform profits, and supply chain profits in **Proposition 6** yields **Proposition 7**.

(a) In Case 1, to ensure that the brand merchant is willing to cooperate under the AI strategy, the transfer payment contract must satisfy conditions $E\pi_{m}^{AI}+F>E\pi_{m}^{RN}$ and $E\pi_{o}^{AI}-F>E\pi_{o}^{RN}$. This leads to conclusions$F_{1\min}=E\pi_{m}^{RN}-E\pi_{m}^{AI}$ and $F_{1\max}=E\pi_{o}^{AI}-E\pi_{o}^{RN}$.

(b) In Case 2, to ensure that the live streaming platform is willing to cooperate under the RI strategy, the transfer payment contract must satisfy conditions$E\pi_{m}^{RI}-F>E\pi_{m}^{RN}$ and $E\pi_{o}^{RI}+F>E\pi_{o}^{RN}$. This leads to conclusions $F_{2\min}=E\pi_{o}^{RN}-E\pi_{o}^{RI}$and $F_{2\max}=E\pi_{m}^{RI}-E\pi_{m}^{RN}$.

(c) In Case 3, to ensure that the live streaming platform is willing to cooperate under the RI strategy, the transfer payment contract must satisfy conditions$E\pi_{m}^{RI}-F>E\pi_{m}^{AI}$and $E\pi_{o}^{RI}+F>E\pi_{o}^{AI}$. This leads to conclusions $F_{3\min}=E\pi_{o}^{AI}-E\pi_{o}^{RI}$ and $F_{3\max}=E\pi_{m}^{RI}-E\pi_{m}^{AI}$.

(d) In Case 4, to ensure that the brand merchant is willing to cooperate under the AI strategy, the transfer payment contract must satisfy conditions $E\pi_{m}^{AI}+F>E\pi_{m}^{RI}$ and$E\pi_{o}^{AI}-F>E\pi_{o}^{RI}$. This leads to conclusions $F_{4\min}=E\pi_{m}^{RI}-E\pi_{m}^{AI}$ and $F_{4\max}=E\pi_{o}^{AI}-E\pi_{o}^{RI}$.

## Proof of Proposition 8

Based on **Proposition 1** and **Proposition 2**,

we obtain$\Delta p^{a}=p^{AI}-p^{RN}$，$\Delta p^{b}=p^{RI}-p^{RN}$，$\Delta p^{c}=p^{RI}-p^{AI}$,$\Delta p^{d}=p^{AI}-p^{RI}$

$\Delta Q_{j}^{a}=Q_{j}^{AI}-Q_{j}^{RN}$,$\Delta Q_{j}^{b}=Q_{j}^{RI}-Q_{j}^{RN}$，$\Delta Q_{j}^{c}=Q_{j}^{RI}-Q_{j}^{AI}$，$\Delta Q_{j}^{d}=Q_{j}^{AI}-Q_{j}^{RI}$.When $E[a|Y]=H$ holds, we derive $\Delta p_{h}^{g}\text{，}\Delta Q_{jh}^{g}\text{，}g=(a,b,c,d)$.When $E[a|Y]=L$ is satisfied, $\Delta p_{l}^{g}\text{，}\Delta Q_{jl}^{g}\text{，}g=(a,b,c,d)$ is obtained.For Case 1, by solving for $\Delta p^{a}=0$, we can determine the threshold value $\beta_{4}=1+\frac{2k(H+L+2Q_{j}^{s}r)(2k+r^{2})}{r^{2}(-2Hk-10kL-12kQ_{j}^{s}r-Hr^{2}+Lr^{2}+2(H-L)(-4k+r^{2})\rho)}$

.When $\beta>\beta_{4}$ is true, $\Delta p^{a}>0$ holds, $\Delta p_{h}^{a}<0,\Delta p_{l}^{a}<0$.When $\beta<\beta_{4}$ is true, $\Delta p^{a}<0$ holds, $\Delta p_{h}^{a}>0,\Delta p_{l}^{a}<0$.

By solving for $\Delta Q_{j}^{a}=0$, we can also determine the threshold value $\beta_{5}=1-\frac{2k(H+L+2Q_{j}^{s}r)}{-(\text{（}H-L\text{）}r^{2}(-1+2\rho))+8k(L+Q_{j}^{s}r+H\rho-L\rho)}$When $\beta>\beta_{5}$ is true, $\Delta Q_{j}^{a}>0$ holds, and then $\Delta Q_{jh}^{a}<0,\Delta Q_{jl}^{a}<0$.When $\beta<\beta_{5}$ holds $\Delta Q_{j}^{a}<0$, $\Delta Q_{jh}^{a}>0,\Delta Q_{jl}^{a}<0$ follows.After performing the calculations, we obtain $\beta_{4}<\beta_{5}$, and, based on the parameter range, we can derive $0<\beta_{4}<\beta_{5}<1$.$\Delta p^{g}\text{，}\Delta Q^{g}\text{，}g=(a,b,c,d)$ can be similarly proved using the method outlined above.Thus, proposition8 is established.

## Proof of Proposition 9

Based on Proposition 1, Proposition 2, and Lemma 3, let $E\pi_{o}^{AI}-E\pi_{o}^{AN}=V_{o}^{AI}$ to obtain

$V_{o}^{AI}=\frac{k^{2}\beta(-(Q_{j}^{s}r+\rho+2(-1+\lambda)\lambda(-1+2\rho))^{2}+\lambda(Q_{j}^{s}r-\frac{1-\rho+\lambda(-1+\rho+H\rho)}{-1+\lambda+\rho-2\lambda\rho})^{2}+(1-\lambda)(Q_{j}^{s}r+\frac{\rho+\lambda(H-(1+H)\rho)}{\lambda+\rho-2\lambda\rho})^{2})}{(2k+r^{2}(-1+\beta))^{2}}$Let$E\pi_{m}^{AI}-E\pi_{m}^{AN}=V_{m}^{AI}$to obtain

$V_{m}^{AI}=\frac{k(-1+\beta)((Q_{j}^{s}r+\rho+2(-1+\lambda)\lambda(-1+2\rho))^{2}-\lambda(Q_{j}^{s}r-\frac{1-\rho+\lambda(-1+\rho+H\rho)}{-1+\lambda+\rho-2\lambda\rho})^{2}+(1-\lambda)(Q_{j}^{s}r+\frac{\rho+\lambda(H-(1+H)\rho)}{\lambda+\rho-2\lambda\rho})^{2})}{4k+2r^{2}(-1+\beta)}$Thus, it follows that if$\frac{1}{2}(r^{2}-r^{2}\beta)<k<1$，then$E\pi_{o}^{AI}>E\pi_{o}^{AN}$,$E\pi_{m}^{AI}>E\pi_{m}^{AN}$

Let$E\pi_{o}^{RI}-E\pi_{o}^{RN}=V_{o}^{RI}$ to obtain

$$V_{m}^{RI}=\frac{\beta(-\lambda(2kQ_{j}^{s}r+r^{2}(\rho+2(-1+\lambda)\lambda(-1+2\rho)+\frac{-1+\lambda+\rho-(1+H)\lambda\rho}{1-\rho+\lambda(-1+2\rho)})+\chi_{1}+\chi_{2}+\chi_{3}+\chi_{4}}{4\text{（}-4k+c)^{2}}$$

Thus, it follows that if$\frac{r^{2}}{4}<k<\frac{r^{2}}{2}$，then $E\pi_{o}^{RI}>E\pi_{o}^{RN}$，if $\frac{r^{2}}{2}<k<1$, then$E\pi_{o}^{RI}<E\pi_{o}^{RN}$

Let$E\pi_{m}^{RI}-E\pi_{m}^{RN}=V_{m}^{RI}$to obtain

$V_{m}^{RI}=\frac{k(-(Q_{j}^{s}r+\rho+2(-1+\lambda)\lambda(-1+2\rho))^{2}+\lambda(Q_{j}^{s}r-\frac{1-\rho+\lambda(-1+\rho+H\rho)}{-1+\lambda+\rho-2\lambda\rho})^{2}+(1-\lambda)(Q_{j}^{s}r+\frac{\rho+\lambda(H-(1+H)\rho)}{\lambda+\rho-2\lambda\rho})^{2})}{8k-2r^{2}}$It is readily observed that when $\frac{r^{2}}{4}<k<1$,$E\pi_{m}^{RI}>E\pi_{m}^{RN}$.

Since，when $\frac{r^{2}}{4}<k<1$, $E\pi_{m}^{RI}>E\pi_{m}^{RN}$,and when $\frac{1}{2}(r^{2}-r^{2}\beta)<k<1$, $E\pi_{m}^{AI}>E\pi_{m}^{AN}$

therefore, by setting $E\pi_{m}^{RI}-E\pi_{m}^{AI}=\Delta E\pi_{m}^{RI-AI}$ to obtain

$\begin{matrix} \Delta E\pi_{m}^{RI-AI}=(4k^{2}(-1+2\beta)((1-\lambda)(1-\rho+\lambda(-1+2\rho))^{2}(\rho+\lambda(H-(1+H)\rho)+Q_{j}^{s}r(\lambda+\rho-2\lambda\rho))^{2} \\ +\lambda(\lambda+\rho-2\lambda\rho)^{2}(1-\rho+\lambda(-1+\rho+H\rho)+Q_{j}^{s}r(1-\rho+\lambda(-1+2\rho)))^{2}))/((8k-2r^{2}) \\ (4k+2r^{2}(-1+\beta))(\lambda+\rho-2\lambda\rho)^{2}(1-\rho+\lambda(-1+2\rho))^{2}) \end{matrix}$.

It is readily observed that when$\frac{1}{2}<\beta<1$and$\frac{r^{2}}{4}<k<\frac{r^{2}}{2}$，$\Delta E\pi_{m}^{RI-AI}>0$，i,e.,$E\pi_{m}^{RI}>E\pi_{m}^{AI}$；when $0<\beta<\frac{1}{2}$and $\frac{r^{2}-r^{2}\beta}{2}<k<\frac{r^{2}}{2}$，$\Delta E\pi_{m}^{RI-AI}<0$，i,e.,$E\pi_{m}^{RI}<E\pi_{m}^{AI}$

Where $\chi_{1}=-2k(\rho+2(-1+\lambda)\lambda(-1+2\rho)+\frac{2(-1+\lambda+\rho-(1+H)\lambda\rho)}{1-\rho+\lambda(-1+2\rho)}))^{2}$

$$\chi_{2}=-(1-\lambda)(2kQ_{j}^{s}r-2k(\rho+2(-1+\lambda)\lambda(-1+2\rho)+\frac{2(\rho+\lambda(H-(-1+H)\rho))}{\lambda+\rho-2\lambda\rho})$$

$$\chi_{3}=r^{2}(\rho+2(-1+\lambda)\lambda(-1+2\rho)-\frac{\rho+\lambda(H-(-1+H)\rho)}{\lambda+\rho-2\lambda\rho}))^{2}$$

$$\chi_{4}=4k^{2}(c(Q_{j}^{s}r-\frac{1-\rho+\lambda(-1+\rho+H\rho)}{-1+\lambda+\rho-2\lambda\rho})^{2}(1-\lambda)(Q_{j}^{s}r+\frac{\rho+\lambda(H-(-1+H)\rho)}{\lambda+\rho-2\lambda\rho})^{2}))$$

## Proof of Proposition 10

The equilibrium solution and the optimal profits of supply chain members under the endogenously determined commission can be derived using the inverse order method based on the profit function in Subsection 6.2.

The equilibrium solution under the brand self-live streaming mode is as follows：

$E\pi_{m}^{AN}=\frac{k(\mathrm{E}[a]+rQ_{j}^{s})^{2}}{16k-8r^{2}}$，$E\pi_{o}^{AN}\frac{k(\mathrm{E}[a]+rQ_{j}^{s})^{2}}{8k-4r^{2}}$，$E\pi_{m}^{AI}=\frac{k(E[a|Y]+rQ_{j}^{s})^{2}}{16k-8r^{2}}$，$E\pi_{o}^{AI}=\frac{k(\mathrm{E}[a|Y]+rQ_{j}^{s})^{2}}{8k-4r^{2}}$

The equilibrium solution under the influential streamer live streaming mode can be proven similarly：

$E\pi_{m}^{RI}=\frac{k(E[a|Y]+rQ_{j}^{s})^{2}}{32k-8r^{2}}$，$E\pi_{o}^{RI}=\frac{k(E[a|Y]+rQ_{j}^{s})^{2}}{16k-4r^{2}}$，$E\pi_{n}^{RI}=\frac{k^{2}(E[a|Y]+rQ_{j}^{s})^{2}}{4(-4k+r^{2})^{2}}$，$E\pi_{m}^{RN}=\frac{6E[a]k+4E[a|Y]k+2krQ_{j}^{s}-ar^{2}+E[a|Y]^{2})^{2}}{32k(4k-r^{2})}$，$E\pi_{o}^{RN}=\frac{(-2(E[a]-2E[a|Y])k+2krQ_{j}^{s}+(E[a]-E[a|Y]+r^{2})^{2}}{16(4k-r^{2})}$，$E\pi_{n}^{RN}=\frac{(-2(E[a]-2E[a|Y])k+2krQ_{j}^{s}+(E[a]-E[a|Y])r^{2})^{2}}{16(-4k+r^{2})^{2}}$

By setting$E\pi_{o}^{AI}-E\pi_{o}^{AN}=V_{o}^{AI}$，we obtain$V_{o}^{AI}=\frac{k(H-L)^{2}(1-2\rho)^{2}}{32k-16r^{2}}$，By setting $E\pi_{m}^{AI}-E\pi_{m}^{AN}=V_{m}^{AI}$，we obtain$V_{m}^{AI*}=\frac{k(H-L)^{2}(1-2\rho)^{2}}{64k-32r^{2}}$. It is readily observed that when $\frac{r^{2}}{2}<k<1$，$E\pi_{o}^{AI}>E\pi_{o}^{AN}$，$E\pi_{m}^{AI}>E\pi_{m}^{AN}$；

By setting$E\pi_{o}^{RI}-E\pi_{o}^{RN}=V_{o}^{RI}$，we obtain $V_{o}^{RI}=-\frac{(H-L)^{2}(12k^{2}-8kr^{2}+r^{4})(1-2\rho)^{2}}{64k(4k-r^{2})^{2}}$，By setting $E\pi_{m}^{RI}-E\pi_{m}^{RN}=V_{m}^{RI}$，we obtain $V_{m}^{RI}=-\frac{(H-L)^{2}(12k^{2}-8kr^{2}+r^{4})(1-2\rho)^{2}}{128k(4k-r^{2})}$. It is readily observed that when $\frac{r^{2}}{2}<k<1$，$E\pi_{o}^{RN}>E\pi_{o}^{RI}$，$E\pi_{m}^{RN}>E\pi_{m}^{RI}$；

By setting$E\pi_{m}^{RI}-E\pi_{m}^{AI}=E\Delta\pi_{m}^{RI-AI}$，we obtain $\Delta E\pi_{m}^{RI-AI}=\frac{k^{2}(-H^{2}-L^{2}-2HQ_{j}^{s}r-2Q_{j}^{s}r(L+Q_{j}^{s}r)+2(H-L)^{2}\rho-2(H-L)^{2}\rho^{2})}{8(8k^{2}-6kr^{2}+r^{4})}$.

It is readily observed that when $\frac{r^{2}}{2}<k<1$，$E\pi_{m}^{RN}>E\pi_{m}^{AI}$，Therefore, the RN mode is selected as the optimal cooperation mode.

## Proof of Proposition 11

The proof process is analogous to that of Proposition 1 and Proposition 2. Thus, the equilibrium solution for competitive brands choosing the brand self-live streaming mode under an imperfectly competitive market can be obtained.

$E\pi_{m_{1}}^{AI}=\frac{E[a|Y]^{2}(-2+b)k+E[a|Y]r^{2}(-1+\beta))^{2}(-1+\beta)}{((-4+b^{2})k-2r^{2}(-1+\beta))^{2}}$，$E\pi_{m_{2}}^{AI}=\frac{(-2+b)^{2}E[a|Y]^{2}k(2k+r^{2}(-1+\beta))(-1+\beta)}{2((-4+b^{2})k-2r^{2}(-1+\beta))^{2}}$，$E\pi_{o}^{AI}=\frac{E[a|Y]^{2}(2(-2+b)^{2}k^{2}+T_{3}}{((-4+b^{2})k-2r^{2}(-1+\beta))^{2}}$，

，，

where，$T_{3}=-2(-2+b)kr^{2}(-1+\beta)+r^{4}(-1+\beta)^{2})\beta$

The equilibrium solution for competitive brands choosing the influential streamer live streaming mode under an imperfectly competitive market can be proven analogously , and is presented as follows:

$E\pi_{m_{1}}^{RI}=\frac{E[a|Y]^{2}((-4+b)k+r^{2})^{2}(-1+\beta)}{((-8+b^{2})k+2r^{2})^{2}}$，$E\pi_{m_{2}}^{RI}=\frac{(-2+b)^{2}E[a|Y]^{2}k(4k-r^{2})}{2((-8+b^{2})k+2r^{2})^{2}}$，$E\pi_{n}^{RI}=-\frac{(-2+b)^{2}E[a|Y]^{2}k(-1+\beta)}{((-8+b^{2})k+2r^{2})^{2}}$，$E\pi_{o}^{RI}=-\frac{E[a|Y]^{2}(2(10+(-6+b)b)k^{2}+T_{7})\beta}{((-8+b^{2})k+2r^{2})^{2}}$，，，，$E\pi_{o}^{RN}=\frac{(((4+b^{2})E[a|Y]^{2}(-4k+r^{2})^{2}+T_{6})\beta)}{4((-8+b^{2})k+2r^{2})^{2}}$

Where $T_{4}=bE[a|Y](-4k+r^{2}))(E[a|Y]k+a(-2+b)r^{2}-bE[a|Y](-4+b^{2})k+r^{2}))^{2}(-1+\beta)$

$T_{5}=E[a|Y](-4k+r^{2}))^{2}(-1+\beta)$, $T_{7}=2(-4+b)kr^{2}+r^{4}$

Subsequently, a comparison is conducted among the equilibrium solutions achieved under information sharing. To guarantee the existence of the optimal solution when information sharing is implemented, the required range of information sharing is specified as：$0<b<b_{1}$and $\frac{r^{2}}{4}<k<k_{3}$or$\frac{r^{2}-r^{2}\beta}{2}<k<k_{4}$,or $b_{1}<b<1$and $\frac{r^{2}}{4}<k<k_{3}$By setting $E\pi_{m_{2}}^{AI}-E\pi_{m_{2}}^{RI}=\frac{(-2+b)^{2}k^{2}(2k^{2}(-32+b^{4}+(-8+b^{2})^{2}\beta)+W_{1}}{4((-8+b^{2})k+2r^{2})^{2}((-4+b^{2})k-2r^{2}(-1+\beta))^{2}}$ to obtained，

It is readily observed that $b\in\left(0,b_{1}\right)$ and $\beta\in\left(\beta_{4}\text{，}\frac{1}{2}\right)$and $k\in\left(\frac{r^{2}-r^{2}\beta}{2},k_{11}\right)$ or $r\in\left(0,r_{1}\right)$ and $b\in\left(0,b_{1}\right]$ and $\beta\in\left(0,\beta_{4}\right)$ and $k\in\left(\frac{r^{2}-r^{2}\beta}{2},k_{12}\right)$，or $\beta\in\left(\beta_{4},\frac{1}{2}\right)$ and $k\in\left(k_{11},k_{12}\right)$，or $r\in\left(r_{1},1\right)$ and $b\in\left(0,b_{1}\right)$ and $\beta\in\left(0,\beta_{4}\right)$ and $k\in\left(\frac{r^{2}-r^{2}\beta}{2},k_{12}\right)$，or $\beta\in\left(\beta_{4},\frac{1}{2}\right)$ and $k\in\left(k_{11},k_{12}\right)$. then $E\pi_{m_{2}}^{RI}>E\pi_{m_{2}}^{AI}$，if $b\in\left(0,b_{1}\right)$ and $k\in\left(k_{12},k_{13}\right)$，or $b\in\left(0,b_{1}\right)$ and $k\in\left(k_{13},1\right)$，then $E\pi_{m_{2}}^{AI}>E\pi_{m_{2}}^{RI}$

where，$k_{3}=-\frac{r^{2}}{-8+b^{2}}$，$k_{4}=\frac{-48r^{2}+128r^{2}\beta+2b^{4}r^{2}\beta-64r^{2}\beta^{2}+16b^{2}r^{2}\beta^{2}-b^{4}r^{2}\beta^{2}}{8(-32+b^{4}+64\beta-16b^{2}\beta+b^{4}\beta)}+S_{2}$，$k_{5}=\frac{-r^{2}+r^{2}\beta}{-4+b^{2}}$，$\beta_{12}=\frac{-4+b^{2}}{-8+b^{2}}$，$b_{1}=8\sqrt{2}\sqrt{\frac{88-20r^{2}+r^{4}}{(32-16r^{2}+r^{4})^{2}}}+\frac{8(-12+r^{2})}{32-16r^{2}+r^{4}}$，$r_{1}=\sqrt{\frac{2}{19+6\sqrt{10}}}$，$\begin{matrix} W_{1}=4r^{4}(-1+\beta)(2+(-4+b^{2})\beta)+kr^{2}(48-2(64-12b^{2}+b^{4})\beta\\ +(-8+b^{2})^{2}\beta^{2}))(1+H^{2}-2(-1+H)^{2}\rho+2(-1+H)^{2}\rho^{2} \end{matrix}$

# Part C. Supplementary Analysis of the Extended Model

In this section, we further examine other extended scenarios that incorporate dimensions such as multiple influential live streamer competition, consumer heterogeneity, dynamic reputation, and platform competition. This is done to test the robustness and universality of the core conclusions of this paper in more complex settings.

## Part C.1 Consumer Heterogeneity

To verify the robustness of the model's conclusions and better characterize the differentiated features of consumers in the real market, this section introduces the setting of consumer heterogeneity to analyze its impact on the brand's cooperation mode selection. Referring to the literature of Zhang et al. [59] and Duan et al. [60], we assume that consumers' base preference for the product is uniformly distributed over the interval [0,1]. Consumer purchase utility differs across the two live streaming modes: the consumer utility function under the brand self-live streaming mode is $u^{A}=\nu+r(Q_{j}+Q_{s})-p$,while the consumer utility function under the influential streamer live streaming mode is $u^{R}=\theta(\nu+r(Q_{j}+Q_{s}))-p$ Here,$\theta(0<\theta<1)$represents the consumer's cognitive discount factor in the influential streamer live streaming scenario, reflecting the consumer's trust in or perceived value of the influential live streamer's recommendation. When $\theta<1$ consumers believe that the influential live streamer's recommendation involves information exaggeration or content distortion, thereby discounting the product's value. Based on the consumer utility maximization principle, their purchase thresholds can be derived: the consumer purchase condition under the brand self-live streaming mode is $\nu\geq p-r(Q_{j}+Q_{s})$and the purchase condition under the influential streamer live streaming mode is $\nu\geq\frac{p}{\theta}-r(Q_{j}+Q_{s})$ Due to the uniform distribution over the interval [0, 1], the corresponding market demands can be expressed as $d^{A}=a-p+r(Q_{j}+Q_{s})$and $d^{R}=a-\frac{p}{\theta}+r(Q_{j}+Q_{s})$，After information sharing, the demands are transformed into $d^{AI}=E[a|Y]-p+r(Q_{j}+Q_{s})$and $d^{RI}=E[a|Y]-\frac{p}{\theta}+r(Q_{j}+Q_{s})$ respectively. The profit function and all other assumptions remain the same as in the basic model. The equilibrium solution and the optimal profits of the supply chain members considering the factor of consumer heterogeneity are obtained using the inverse order method. Combining this with Lemma 1, we derive the live streaming e-commerce platform's information sharing strategy. Further combining the platform's information sharing strategy, the brand's equilibrium cooperation mode selection can be determined by comparing the brand's optimal profit levels under the brand self-live streaming mode and the influential streamer live streaming mode. This leads to Proposition A.1.

Proposition A.1 When $\frac{2-\theta}{2}<\beta<1$and$\frac{r^{2}\theta}{4}<k<\frac{r^{2}\theta}{2}$, the brand's equilibrium strategy combination is the RI mode; when $1-\theta<\beta<\frac{2-\theta}{2}$and $\frac{r^{2}-r^{2}\beta}{2}<k<\frac{r^{2}\theta}{2}$, the brand's equilibrium strategy combination is the AI mode.

In the extended model that considers the impact of consumer heterogeneity, the emergence of AI (brand self-live streaming with information sharing) and RI (influential streamer live streaming with information sharing) as the optimal cooperation modes reflects the profound influence of consumer cognitive differences on the strategy choices of the brand and the platform.Specifically, the introduction of the consumer cognitive discount factor indicates that some consumers have a higher level of trust or perceived value regarding the influential live streamer's recommendation. Consequently, the influential streamer live streaming mode (RI), when $\theta$ is high (i.e., consumer trust in the influential live streamer is strong), can effectively enhance the demand responsiveness $r$ through information sharing. This achieves a higher marginal return on traffic data investment, thus becoming the optimal choice within the medium traffic data cost range $\frac{r^{2}\theta}{4}<k<\frac{r^{2}\theta}{2}$. Conversely, when $\theta$ is low (i.e., consumers have higher trust in brand self-live streaming information), the brand self-live streaming mode (AI), supported by information sharing, can mitigate the risks arising from demand uncertainty. Furthermore, by making precise traffic data investments $\frac{r^{2}-r^{2}\beta}{2}<k<\frac{r^{2}\theta}{2}$ , it enhances its own profit performance.Compared to the basic model, the results of the extended model suggest that consumer cognitive differences introduce a "trust effect," causing the boundaries of the optimal cooperation mode to depend not only on the relative relationship between the traffic data cost $k$ and the platform commission rate $\beta$, but also on the consumers' perceived value of different live streaming entities.Therefore, consumer heterogeneity makes the optimal ranges for the AI and RI modes more flexible, reflecting the critical moderating role of the interaction between trust and information sharing in the selection of cooperation modes within the digital consumption environment.

## Part C.2 Multi-Influential Streamer Competition

In the basic model, it is assumed that only a single influential live streamer exists in the market, resulting in a relatively simple interaction between the platform and the brand. However, in the real live streaming e-commerce ecosystem, there are numerous influential live streamers, and significant audience overlap and traffic data competition exist among different streamers. This forces the brand to face more complex strategic trade-offs when selecting a cooperation mode. Therefore, this section extends the model to the case of multi-influential live streamer competition to further verify the robustness of the basic model's conclusions.

Referring to the research of Dong et al. [63], the basic model considers the brand's selection of a cooperation mode with a single influential live streamer. This section will consider the brand's cooperation mode selection when influential live streamer competition exists under information sharing. Assume there are two influential live streamers in the market, denoted by $n_{1},n_{2}$.They have a certain degree of cross-influence among the consumer group, and their competition level is represented by the parameter $b_{n}(0<b_{n}<1)$.When $b_{n}$ increases, it indicates a higher degree of audience overlap between the streamers and more intense competition. Under information sharing, the brand possesses demand data from the platform and can adjust its traffic data investments and cooperation mode based on real-time market feedback. At this point, two possible cooperation modes are considered: the brand self-live streaming mode and the influential streamer live streaming mode.

Specifically, considering demand information sharing, the brand $m$resells the product to influential live streamer (denoted by $n_{1}$) at a wholesale price $\omega_{1}$. Influential live streamer $n_{1}$ then sells the product to consumers through the platform. Streamer $n_{1}$decides on the sales quantity $q_{1}$ and $\beta$ pays to the platform. To increase sales and expand the live streaming e-commerce channel, the brand may operate under the brand self-live streaming mode, selling the product directly to consumers through the platform. In this case, $m$ decides on the sales quantity $q_{2}$ and pays a commission rate of $\beta$ to the platform. If $m$ operates under the influential streamer live streaming mode, then $m$ will resell the product to the other influential live streamer (denoted by $n_{2}$ ) at a wholesale price $\omega_{2}$. Influential live streamer $n_{2}$ sells the product to consumers through the platform. At this time, streamer $n_{2}$ decides on the sales quantity and pays a commission rate of $\beta$to the platform. Simultaneously, the brand purchases commercial traffic data to $Q_{j}$ input in consumers, thereby increasing product exposure and enhancing market demand.

The inverse demand function of the market is $p_{1}=a-q_{1}-b_{n}q_{2}+r(Q_{j}+Q_{j}^{s})$and$p_{2}=a-q_{2}-b_{n}q_{1}+r(Q_{j}+Q_{j}^{s})$When the brand chooses to operate under the brand self-live streaming mode, the profit functions for the brand and the platform are

$E\pi_{m}^{AI}=\omega_{1}q_{1}+(1-\beta)E[p_{2}q_{2}|Y]-\frac{1}{2}kQ_{j}^{2}$，$E\pi_{n_{1}}^{AI}=(1-\beta)E[(p_{1}-\omega_{1})q_{1}|Y]$，$E\pi_{o}^{AI}=\beta E[(p_{1}-\omega_{1})q_{1}|Y]+\beta E[p_{2}q_{2}|Y]$，$E\pi_{m}^{AN}=\omega_{1}q_{1}+(1-\beta)E[p_{2}q_{2}]-\frac{1}{2}kQ_{j}^{2}$，$E\pi_{n_{1}}^{AN}=(1-\beta)E[(p_{1}-\omega_{1})q_{1}]$，$E\pi_{o}^{AN}=\beta E[(p_{1}-\omega_{1})q_{1}]+\beta E[p_{2}q_{2}]$

When the brand chooses to operate under the influential streamer live streaming mode, the profit function are

$E\pi_{m}^{RN}=E[\omega_{1}q_{1}+\omega_{2}q_{2}]-\frac{1}{2}kQ_{j}^{2}$,$E\pi_{n_{1}}^{RN}=(1-\beta)E[(p_{1}-\omega_{1})q_{1}|Y]$,$E\pi_{n_{2}}^{RN}=(1-\beta)E[(p_{2}-\omega_{2})q_{2}|Y]$,$E\pi_{o}^{RN}=\beta E[(p_{1}-\omega_{1})q_{1}|Y]+\beta E[(p_{2}-\omega_{2})q_{2}|Y]$，$E\pi_{m}^{RI}=E[\omega_{1}q_{1}+\omega_{2}q_{2}|Y]-\frac{1}{2}kQ_{j}^{2}$,$E\pi_{n_{1}}^{RI}=(1-\beta)E[(p_{1}-\omega_{1})q_{1}|Y]$,$E\pi_{n_{2}}^{RN}=(1-\beta)E[(p_{2}-\omega_{2})q_{2}|Y]$ $E\pi_{o}^{RN}=\beta E[(p_{1}-\omega_{1})q_{1}|Y]+\beta E[(p_{2}-\omega_{2})q_{2}|Y]$

To ensure the existence of an internal optimal solution, we assume that the commission rate $\beta$satisfies the constraint $0<\beta<1/2$，, which is a common assumption in the literature [40].

The equilibrium solution and the optimal profits of the supply chain members considering the factor of multi-influential live streamer competition are obtained using the inverse order method. Combining this with Lemma 1, we derive the live streaming e-commerce platform's information sharing strategy. Further combining the platform's information sharing strategy, the brand's equilibrium cooperation mode selection can be determined by comparing the brand's optimal profit levels under the brand self-live streaming mode and the influential streamer live streaming mode. This leads to Proposition A.2.

Proposition A.2 When$\frac{r^{2}}{2+b_{n}}<k<\frac{2r^{2}}{2+b_{n}}$and$\beta_{13}<\beta<\frac{1}{2}$，he brand's equilibrium strategy combination is the RI mode；when$\frac{r^{2}}{2+b_{n}}<k<k_{6}$and$\beta_{14}<\beta<\beta_{13}$，or $k_{6}<k<\frac{2r^{2}}{2+b_{n}}$and$0<\beta<\beta_{13}$ the brand's equilibrium strategy combination is the AI mode.

where $\beta_{13}=\frac{-8+4b-b_{n}^{3}}{-16+2b_{n}^{2}}$，$\beta_{14}=\frac{-16k+6b_{n}^{2}k+12r^{2}-8b_{n}r^{2}+b_{n}^{2}r^{2}}{2b_{n}^{2}k+8r^{2}-4b_{n}r^{2}}$

In the extended model of multi-influential live streamer competition, the competition coefficient among the streamers introduces an internal competition effect between channels, leading to diminishing marginal returns for influential streamer live streaming. When competition is intense, streamers increase their marketing investment to vie for limited consumer attention. This intensifies the platform commission's squeezing effect on streamer profits, motivating the brand to more strongly prefer taking the initiative through brand self-live streaming (AI mode) and reducing the uncertainty of profits caused by relying on external streamers. Secondly, under information sharing, the transparency of demand information increases the marginal benefit of traffic data investment. Both the brand and the platform can allocate traffic data based on more precise demand expectations, thereby enhancing the overall efficiency of the AI and RI modes within a certain range. Furthermore, compared to the results from the basic model with only a single streamer or no competition, the extended model demonstrates that the existence of competition highlights the importance of information sharing—it not only alleviates resource waste under multi-agent competition but also enhances demand matching. Consequently, under moderate traffic data cost and commission rates, the information sharing modes (AI and RI) gain a comparative advantage over the non-sharing mode (RN). Overall, multi-influential live streamer competition weakens the advantage of influential streamer live streaming in the non-information sharing context and strengthens the economic rationale for brand self-live streaming and the information sharing mechanism in maximizing market efficiency and profit.

## Part C.3 Influence of Streamer Reputation Dynamics

In analyzing the optimal cooperation modes for the brand and the platform under conditions of information sharing and non-sharing, the basic model did not account for the influence of streamer characteristics on consumer decision-making and market demand. However, in practical live streaming e-commerce scenarios, the streamer's reputation (e.g., credibility, influence, fan loyalty, etc.) significantly impacts consumer purchase intention and brand perception, potentially altering the optimal decisions of all parties. Therefore, this section extends the research to consider the scenario of streamer reputation heterogeneity to further verify the robustness of the basic model's conclusions.

Referring to the literature of Peng [64], let the streamer's reputation be represented by the parameter $\gamma_{i}$,$i\in\{m,n\}$,where$\gamma_{m}(0<\gamma_{m}<1)$denotes the reputation level of the brand’s own streamer (amateur streamer), and $\gamma_{n}(0<\gamma_{n}<1)$ represents the reputation level of the influential live streamer. A higher reputation signifies stronger consumer trust and higher traffic data conversion efficiency, which can influence market demand through a signaling effect. Therefore, $0<\gamma_{m}<\gamma_{n}$. When streamer reputation is considered, the market demand for the product can be expressed as $d=\gamma_{i}a-\eta p+r(Q_{j}+Q_{j}^{s})$ where $\eta(0<\eta<1)$is the price sensitivity coefficient. The profit function and all other assumptions are the same as in the basic model. The game equilibrium solution and the optimal profits of the supply chain members considering streamer reputation dynamics are obtained using the inverse order method. We further analyze the brand's optimal cooperation mode selection. To ensure the existence of the optimal solution, the information sharing interval is set to $\frac{r^{2}}{4}<\eta<1$ and $\frac{r^{2}}{4\eta}<k<1$ This leads to Proposition A.3.

Proposition A.3 When$\eta=\frac{r^{2}}{2}$ and $1-\beta<k<1$ and $0<\gamma_{m}<\gamma_{m_{1}}$ and $0<Q_{j}^{s}<Q_{j_{1}}^{s}$ or when$\eta=\frac{r^{2}}{2}$and $\frac{1}{2}<\beta<1$ and $\frac{1}{2}<k<1$, RI is the optimal cooperation mode; and when $\eta=\frac{r^{2}}{2}$and $0<\gamma_{m}<\gamma_{m_{1}}$ and $Q_{j}^{s}>Q_{j_{1}}^{s}$or when $\eta=\frac{r^{2}}{2}$ and $\gamma_{m_{1}}<\gamma_{m}<\gamma_{n}$, AI is the optimal cooperation mode.

where，$\gamma_{m_{1}}=\sqrt{\frac{\gamma_{n}^{2}-k\gamma_{n}^{2}-\beta\gamma_{n}^{2}}{(-1+2k)(-1+\beta)}}$,$Q_{j_{1}}^{s}=\frac{1}{2}(\frac{(H+L)((-1-2k(-1+\beta)+\beta)\gamma_{m}+\gamma_{n}-(k+\beta)\gamma_{n})}{kr(-1+2\beta)}+L_{1}$

When streamer reputation is introduced, the reason for the emergence of AI (brand self-live streaming with information sharing) and RI (influential streamer live streaming with information sharing) as the optimal cooperation modes primarily lies in the structural impact of the reputation variable on market signal transmission and the marginal return of traffic data under conditions of demand information asymmetry. In the basic model, the brand and the platform's optimal cooperation mode selection is mainly determined by the interplay of traffic data investment cost, traffic data conversion rate, and commission rate—that is, market efficiency is determined solely by cost and the information sharing mechanism. However, the introduction of the streamer reputation parameter in the extended model alters this equilibrium condition.

Specifically, an influential live streamer with a high reputation can influence consumer perception and amplify the value of information sharing, thereby improving the platform's traffic data conversion rate. This causes the RI mode to outperform other modes within the range of higher commission rates or moderate traffic data costs. Conversely, when the brand's own streamer has a high reputation, information sharing reinforces the brand signal effect of its self-live streaming, increasing the marginal return of traffic data investment, making the AI mode the optimal choice under certain parameter ranges. Compared to the basic model, where mode selection is determined solely by a cost-benefit trade-off, the extended model reveals that the reputation mechanism reshapes the optimal cooperation mode boundaries in live streaming e-commerce by altering information efficiency and the trust structure: specifically, when the reputation difference is sufficient to compensate for the efficiency loss caused by information asymmetry, the information sharing modes (AI and RI) exhibit a greater economic advantage than the non-sharing mode (RN).

## Part C.4 Multi-Platform Competition

In the basic model, it is assumed that only a single live streaming e-commerce platform exists in the market, leading to a relatively stable relationship between the platform and the brand. However, with the rapid development of the live streaming e-commerce ecosystem, brands often maintain agency partnerships with retail e-commerce platforms while further expanding their live streaming channels, achieving traffic data conversion and market penetration through both brand self-live streaming and influential streamer live streaming modes. To verify the robustness of the basic model's conclusions, and referring to the literature of Zhao et al. [61] and Zhang et al. [62], this section introduces an extended model of multi-platform competition to characterize the brand’s optimal decision-making behavior in a multi-platform competitive environment.

Specifically, assume there are two types of e-commerce platforms in the market, denoted by$o_{1},o_{2}$,They have a certain degree of cross-influence among the consumer group, and their competition level is represented by the parameter $b_{o}(0<b_{o}<1)$. When $b_{o}$ increases, it indicates a higher degree of audience overlap between the platforms and more intense competition. Under information sharing, the brand possesses demand data from the platforms and can adjust its traffic data investments and cooperation mode based on real-time market feedback.

At this point, the brand $m$ cooperates with the retail e-commerce platform (denoted by $o_{1}$) via an agency model: the brand sells the product to consumers on the platform at a retail price of $p_{1}$ with a sales quantity of $q_{1}$ and pays a proportional commission of $\beta$ to the platform. Simultaneously, the brand expands on the live streaming e-commerce platform. If the brand operates under the brand self-live streaming mode, it sells the product to consumers through the platform. Then $m$determines the sales price $p_{2}$and sales quantity $q_{2}$ and pays $\beta$ to the platform. If the brand operates under the influential streamer live streaming mode, then $m$ will resell the product to the influential live streamer (denoted by $n$) at a wholesale price $\omega$.Influential live streamer $n$ sells the product to consumers through the platform. At this time, the streamer $n$ determines the sales price $p_{2}$and sales quantity $q_{2}$ and pays a proportional commission of $\beta$ to the platform. The inverse demand functions for the two sales channels are assumed to be $p_{1}=a-q_{1}-b_{o}q_{2}$, $p_{2}=a-q_{2}-b_{o}q_{1}+rQ_{j}$ respectively. The expected profit functions for the supply chain members under the brand self-live streaming mode can be obtained.

$E\pi_{m}^{AN}=(1-\beta)E[p_{1}q_{1}]+(1-\beta)E[p_{2}q_{2}]-\frac{1}{2}kQ_{j}^{2}$，$E\pi_{o_{1}}^{AN}=\beta E[p_{1}q_{1}]$ $E\pi_{o_{2}}^{AN}=\beta E[p_{2}q_{2}]$

$E\pi_{m}^{AI}=(1-\beta)E[p_{1}q_{1}|Y]+(1-\beta)E[p_{2}q_{2}|Y]-\frac{1}{2}kQ_{j}^{2}$，$E\pi_{o_{1}}^{AI}=\beta E[p_{1}q_{1}|Y]$，$E\pi_{o_{2}}^{AI}=\beta E[p_{2}q_{2}|Y]$

The expected profit functions for the supply chain members under the influential streamer live streaming mode are as follows:

$E\pi_{m}^{RN}=(1-\beta)E[p_{1}q_{1}]+\omega E[q_{2}]-\frac{1}{2}kQ_{j}^{2}$，$E\pi_{n}^{RN}=(1-\beta)+E[(p_{2}-\omega)q_{2}|Y]$，$E\pi_{o_{1}}^{RN}=\beta E[p_{1}q_{1}]$ $E\pi_{o_{2}}^{RN}=\beta E[(p_{2}-\omega)q_{2}|Y]$，$E\pi_{m}^{RI}=(1-\beta)E[p_{1}q_{1}|Y]+E[\omega q_{2}|Y]-\frac{1}{2}kQ_{j}^{2}$

$E\pi_{n}^{RI}=(1-\beta)+E[(p_{2}-\omega)|Y]q_{2}-\frac{1}{2}kQ_{j}^{2}$，$E\pi_{o_{1}}^{RI}=\beta E[p_{1}q_{1}|Y]$ $E\pi_{o_{2}}^{RI}=\beta E[(p_{2}-\omega)q_{2}|Y]$

To ensure the existence of an internal optimal solution, we assume that the commission rate $\beta$ satisfies the constraint $0<\beta<1/2$. which is a common assumption in the literature [40]. The equilibrium solution and the optimal profits of the supply chain members considering the factor of multi-platform competition are obtained using the inverse order method. Combining this with Lemma 1, we derive the live streaming e-commerce platform's information sharing strategy. Further combining the platform's information sharing strategy, the brand's equilibrium cooperation mode selection can be determined by comparing the brand's optimal profit levels under the brand self-live streaming mode and the influential streamer live streaming mode. As a closed-form solution is not available for the comparison of brand profits under different information sharing modes, we verified the robustness of the basic model through numerical experiments. The parameter values, corresponding equilibrium commission rates, and equilibrium strategy combinations used in the numerical experiments are detailed in Table A.2 in the part D of Appendix .This leads to Proposition A.4.

Proposition A.4 When the commission rate $\beta$ and the traffic data conversion rate $r$ are high, the brand's optimal cooperation mode is RI. When the commission rate $\beta$, the traffic data conversion rate $r$ and the platform competition intensity are low, the brand's optimal cooperation mode is AI.

The numerical simulation results of the extended model reveal a novel decision logic for the brand's live streaming cooperation mode selection, which is fundamentally different from the theoretical conclusions of the basic model based on critical parameter values. The largest distinction is that, upon introducing the platform competition coefficient ($b$) and the demand signal ($\rho$) into the model, platform competition intensity replaces the complex relationship between $\beta$ and$r^{2}/k$ from the basic model as the primary driving force behind mode selection. Specifically, a high-competition environment (high $b$) strongly leads to influential streamer live streaming (RI), while a low-competition environment (low$b$) leads to brand self-live streaming (AI). Furthermore, all equilibrium modes in the numerical simulation are based on information sharing, causing the information non-sharing RN mode from the basic model to completely disappear, thereby underscoring the criticality of information value in a competitive market. This shift indicates that the brand's decision-making is no longer merely a game of internal costs and incentives but an adaptation to the external competitive environment.

Based on the numerical simulation analysis of the extended model, we can clearly observe the brand's optimal strategy for live streaming cooperation mode selection. Platform competition intensity and traffic data conversion efficiency are the decisive factors: In a market environment characterized by high competition and high commission rates, complemented by a high traffic data conversion rate, the influential streamer live streaming (RI) mode can generate positive and substantial profits for the brand, making it the optimal profitability strategy. This highlights the importance of leveraging the influential live streamer's professional capability and high conversion efficiency amid fierce competition. Conversely, the brand self-live streaming (AI) mode frequently appears under combinations of low competition and low efficiency, positioning it more as a defensive or fallback strategy adopted by the brand when market conditions are suboptimal. Therefore, the managerial implication for the brand is to regard information sharing as a rigid requirement and use the platform competition coefficient as the primary determinant. When facing high competition, the brand should actively seek collaboration with influential live streamers that offer high conversion rates; even if this entails bearing a higher commission, the priority must be ensuring efficiency and profitability. In a low-competition environment, however, the brand must strictly control costs and use brand self-live streaming as a means of capability cultivation and maintenance of operations.

# Part D. Proof for the Supplementary Analysis of the Extended Model

## Proof of Proposition A.1

The equilibrium solution considering the factor of consumer heterogeneity is presented as follows:

$E\pi_{m}^{AI}=\frac{k(E[a|Y]+rQ_{j}^{s})^{2}(-1+\beta)}{4k+2r^{2}(-1+\beta)}$,$E\pi_{o}^{AI}=\frac{k^{2}(E[a|Y]+rQ_{j}^{s})^{2}\beta}{(2k+r^{2}(-1+\beta))^{2}}$，，$E\pi_{o}^{AN}=\frac{k^{2}(a+rQ_{j}^{s})^{2}\beta}{(2k+r^{2}(-1+\beta))^{2}}$，$E\pi_{m}^{RI}=\frac{k(E[a|Y]+rQ_{j}^{s})^{2}\theta}{8k-2r^{2}\theta}$，$E\pi_{o}^{RI}=\frac{k^{2}(E[a|Y]+rQ_{j}^{s})^{2}\beta\theta}{(-4k+r^{2}\theta)^{2}}$，$E\pi_{n}^{RI}=-\frac{k^{2}(E[a|Y]+rQ_{j}^{s})^{2}(-1+\beta)\theta}{(-4k+r^{2}\theta)^{2}}$，，，

By setting $E\pi_{o}^{AI}-E\pi_{o}^{AN}=V_{o}^{AI*}$，$E\pi_{m}^{AI}-E\pi_{m}^{AN}=V_{m}^{AI*}$，we obtained $V_{o}^{AI}=\frac{k^{2}(H-L)^{2}\beta(1-2\rho)^{2}}{4(2k+r^{2}(-1+\beta))^{2}}$，$V_{m}^{AI}=\frac{k^{2}(H-L)^{2}(-1+\beta)(1-2\rho)^{2}}{8(2k+r^{2}(-1+\beta))}$. It is readily observed that when，$\frac{r^{2}-r^{2}\beta}{2}<k<1$，$E\pi_{o}^{AI}>E\pi_{o}^{AN}$，$E\pi_{m}^{AI}>E\pi_{m}^{AN}$。

By setting$E\pi_{o}^{RI}-E\pi_{o}^{RN}=V_{o}^{RI}$，we obtained $V_{o}^{RI}=-\frac{(H-L)^{2}\beta\theta(12k^{2}-8kr^{2}\theta+r^{4}\theta^{2})(1-2\rho)^{2}}{16(-4k+r^{2}\theta)^{2}}$. It is readily observed that when$\frac{r^{2}\theta}{4}<k<\frac{r^{2}\theta}{2}$, $E\pi_{o}^{RI}>E\pi_{o}^{RN}$；when $\frac{r^{2}\theta}{2}<k<1$，$E\pi_{o}^{RI}<E\pi_{o}^{RN}$；

By setting$E\pi_{m}^{RI}-E\pi_{m}^{RN}=V_{m}^{RI}$, we obtained $V_{m}^{RI}=\frac{k(H-L)^{2}\theta(1-2\rho)^{2}}{32k-8r^{2}\theta}$，It is readily observed that when $\frac{r^{2}\theta}{4}<k<1$, $E\pi_{m}^{RI}>E\pi_{m}^{RN}$。

By setting $E\pi_{m}^{RI}-E\pi_{m}^{AI}=\Delta E\pi_{m}^{RI-RI}$, we obtained

. It is readily observed that when $\frac{2-\theta}{2}<\beta<1$and $\frac{r^{2}\theta}{4}<k<\frac{r^{2}\theta}{2}$,$E\pi_{m}^{RI}>E\pi_{m}^{AI}$，when $1-\theta<\beta<\frac{2-\theta}{2}$and $\frac{r^{2}-r^{2}\beta}{2}<k<\frac{r^{2}\theta}{2}$，$E\pi_{m}^{RI}<E\pi_{m}^{AI}$

## Proof of Proposition A.2

The equilibrium solution considering the factor of multi-influential live streamer competition is presented as follows:

$E\pi_{m}^{AI}=\frac{(-2+b_{n})k(E[a|Y]+Q_{j}^{s}r)^{2}(-6+b_{n}+4\beta)}{4k(8+b_{n}^{2}(-3+\beta))-2(-2+b_{n})r^{2}(-6+b_{n}+4\beta)}$，$E\pi_{o}^{AI}=\frac{k^{2}(E[a|Y]+Q_{j}^{s}r)^{2}\beta(80+b_{n}(16-4+\beta)+b(4+b_{n}(4+b_{n})+4(-4+\beta)\beta)))}{(-2k(8+b_{n}^{2}(-3+\beta))+(-2+b_{n})r^{2}(-6+b_{n}+4\beta))^{2}}$

$E\pi_{n_{1}}^{AI}=\frac{4k^{2}(E[a|Y]+Q_{j}^{s}r)^{2}(2+b_{n}(-2+\beta))^{2}(-1+\beta)}{(-2k(8+b_{n}^{2}(-3+\beta))+(-2+b_{n})r^{2}(-6+b_{n}+4\beta))^{2}}$，

，

,,

，，

，

$$E\pi_{o}^{RI}=\frac{k^{2}(E[a|Y]+Q_{j}^{s}r)^{2}\beta}{2(-((2+b_{n})k)-r^{2})^{2}}$$

$$E\pi_{m}^{RI}=\frac{k^{2}(E[a|Y]+Q_{j}^{s}r)^{2}}{2(2+b_{n})k-2r^{2}}$$

$$E\pi_{n_{1}}^{RI}=\frac{k^{2}(E[a|Y]+Q_{j}^{s}r)^{2}(-1+\beta)}{4(-((2+b_{n})k)+r^{2})^{2}}$$

$$E\pi_{n_{2}}^{RI}=\frac{k^{2}(E[a|Y]+Q_{j}^{s}r)^{2}(-1+\beta)}{4(-((2+b_{n})k)+r^{2})^{2}}$$

By setting$E\pi_{o}^{AI}-E\pi_{o}^{AN}=V_{o}^{AI}$，$E\pi_{m}^{AI}-E\pi_{m}^{AN}=V_{m}^{AI}$we obtained $V_{o}^{AI}=\frac{k^{2}(H-L)^{2}\beta(80+b_{n}(16(-4+\beta)+b_{n}(4+b_{n}(4+b_{n})+4(-4+\beta)\beta)))(1-2\rho)^{2}}{4(-2k(8+b_{n}^{2}(-3+\beta))+(-2+b_{n})r^{2}(-6+b_{n}+4\beta))^{2}}$，

$V_{m}^{AI}=\frac{(-2+b_{n})k(H-L)^{2}(-6+b_{n}+4\beta)(1-2\rho)^{2}}{16k(8+b_{n}^{2}(-3+\beta))-8(-2+b_{n})r^{2}(-6+b_{n}+4\beta)}$. It is readily observed that when$k_{6}<k<1$，$E\pi_{o}^{AI}>E\pi_{o}^{AN}$，$E\pi_{m}^{AI}>E\pi_{m}^{AN}$, where $k_{6}=\frac{12r^{2}-8b_{n}r^{2}+b_{n}^{2}r^{2}-8r^{2}\beta+4b_{n}r^{2}\beta}{16-6b_{n}^{2}+2b_{n}^{2}\beta}$，

By setting $E\pi_{o}^{RI}-E\pi_{o}^{RN}=V_{o}^{RI}$，we obtained $V_{o}^{RI}=\frac{(H-L)^{2}(3(2+b_{n})^{2}k^{2}-8(2+b_{n})kr^{2}+4r^{4})\beta(1-2\rho)^{2}}{8(2+b_{n})^{2}(-((-2+b_{n})k)+r^{2})^{2}}$，

It is readily observed that when$\frac{r^{2}}{2+b_{n}}<k<\frac{2r^{2}}{2+b_{n}}$，$E\pi_{o}^{RI}>E\pi_{o}^{RN}$，when$\frac{2r^{2}}{2+b_{n}}<k<1$，$E\pi_{o}^{RI}<E\pi_{o}^{RN}$.

By setting$E\pi_{m}^{RI}-E\pi_{m}^{RN}=V_{m}^{RI}$，we obtained $V_{m}^{RI*}=\frac{k(H-L)^{2}(1-2\rho)^{2}}{8(2+b_{n})k-8r^{2}}$ It is readily observed that when $\frac{2r^{2}}{2+b_{n}}<k<1$, $E\pi_{m}^{RI}>E\pi_{m}^{RN}$.

By setting$E\pi_{m}^{RI}-E\pi_{m}^{AI}=\Delta E\pi_{m}^{RI-AI}$，$\Delta E\pi_{m}^{RI-AI}=\frac{1}{2}k(\frac{1}{2(2+b_{n})k-2r^{2}}-\frac{(-2+b_{n})(-6+b_{n}+4\beta)}{4k(8+b_{n}^{2}(-3+\beta))-2(-2+b_{n})r^{2}(-6+b_{n}+4\beta)})((L+Q_{j}^{s}r+H\rho-L\rho)^{2}+(H+Q_{j}^{s}r-H\rho+L\rho)^{2}$

It is readily observed that when$\frac{r^{2}}{2+b_{n}}<k<\frac{2r^{2}}{2+b_{n}}$and $\beta_{13}<\beta<\frac{1}{2}$, $E\pi_{m}^{RI}>E\pi_{m}^{AI}$.

When $\frac{r^{2}}{2+b_{n}}<k<k_{21}$and $\beta_{14}<\beta<\beta_{13}$，or $k_{21}<k<\frac{2r^{2}}{2+b_{n}}$and $0<\beta<\beta_{13}$，$E\pi_{m}^{RI}<E\pi_{m}^{AI}$.

Where $\beta_{13}=\frac{-8+4b-b_{n}^{3}}{-16+2b_{n}^{2}}$,$\beta_{14}=\frac{-16k+6b_{n}^{2}k+12r^{2}-8b_{n}r^{2}+b_{n}^{2}r^{2}}{2b_{n}^{2}k+8r^{2}-4b_{n}r^{2}}$,$k_{6}=\frac{12r^{2}-8b_{n}r^{2}+b_{n}^{2}r^{2}-8r^{2}\beta+4b_{n}^{2}r^{2}\beta}{16-6b_{n}^{2}+2b_{n}^{2}\beta}$.

## Proof of Proposition A.3

The equilibrium solution considering the factor of streamer reputation dynamics is presented as follows:

，，$E\pi_{o}^{AI}=\frac{k^{2}\beta(Q_{j}^{s}r+E[a|Y]\gamma_{m})^{2}\eta}{(r^{2}(-1+\beta)+2k\eta}$，$E\pi_{m}^{AI}=-\frac{k^{2}(-1+\beta)(Q_{j}^{s}r+E[a|Y]\gamma_{m})^{2}}{2r^{2}(-1+\beta)+4k\eta}$，，，

，$E\pi_{m}^{RI}=\frac{k(Q_{j}^{s}r+E[a|Y]\gamma_{n})^{2}}{-r^{2}+4k\eta}$，$E\pi_{o}^{RI}=\frac{k^{2}\beta(Q_{j}^{s}r+E[a|Y]\gamma_{n})^{2}\eta}{(r^{2}-4k\eta)^{2}}$，$E\pi_{n}^{RI}=-\frac{k^{2}(-1+\beta)(Q_{j}^{s}r+E[a|Y]\gamma_{n})^{2}\eta}{(r^{2}-4k\eta)^{2}}$.

By setting$E\pi_{o}^{AI}-E\pi_{o}^{AN}=V_{o}^{AI}$, we obtained $V_{o}^{AI}=\frac{k^{2}(H-L)^{2}\beta\gamma_{m}^{2}\eta(1-2\rho)^{2}}{4(r^{2}(-1+\beta)+2k\eta)^{2}}$. It is readily observed that when $0<\eta<\frac{r^{2}}{2}$and$\frac{r^{2}-2\eta}{r^{2}}<\beta<1$and$\frac{r^{2}-r^{2}\beta}{2\eta}<k<1$，or $\frac{r^{2}}{2}\leq\eta<1$and $\frac{r^{2}-r^{2}\beta}{2\eta}<k<1$ , $E\pi_{o}^{AI}>E\pi_{o}^{AN}$.

By setting$E\pi_{m}^{AI}-E\pi_{m}^{AN}=V_{m}^{AI}$, we obtained $V_{m}^{AI}=\frac{k(H-L)^{2}(1-\beta)\gamma_{m}^{2}(1-2\rho)^{2}}{8(r^{2}(-1+\beta)+2k\eta)}$. It is readily observed that when $0<\eta<\frac{r^{2}}{2}$and $\frac{r^{2}-2\eta}{r^{2}}<\beta<1$and $\frac{r^{2}-r^{2}\beta}{2\eta}<k<1$，or $\frac{r^{2}}{2}\leq\eta<1$ and $\frac{r^{2}-r^{2}\beta}{2\eta}<k<1$, $E\pi_{m}^{AI}>E\pi_{m}^{AN}$.

By setting $E\pi_{o}^{RI}-E\pi_{o}^{RN}=V_{o}^{RI}$, we obtained $V_{o}^{RI*}=\frac{(H-L)^{2}\beta\gamma_{n}^{2}(r^{2}-6k\eta)(r^{2}-2k\eta)(1-2\rho)^{2}}{16\eta(r^{2}-4k\eta)^{2}}$. It is readily observed that when $\frac{r^{2}}{4}<\eta\leq\frac{r^{2}}{2}$and $\frac{r^{2}}{4\eta}<k<1$ or $\frac{r^{2}}{2}<\eta<1$ and $\frac{r^{2}}{4\eta}<k<\frac{r^{2}}{2\eta}$，$E\pi_{o}^{RI}>E\pi_{o}^{RN}$；when $\frac{r^{2}}{2}<\eta<1$ and $\frac{r^{2}}{2\eta}<k<1$，$E\pi_{o}^{RI}<E\pi_{o}^{RN}$.

By setting $E\pi_{m}^{RI}-E\pi_{m}^{RN}=V_{m}^{RI}$ we obtained $V_{m}^{AI}=\frac{k(H-L)^{2}\gamma_{n}^{2}(1-2\rho)^{2}}{8(r^{2}-4k\eta)}$. It is readily observed that when $\frac{r^{2}}{4}<\eta\leq\frac{r^{2}}{2}$and $\frac{r^{2}}{4\eta}<k<1$or $\frac{r^{2}}{2}<\eta<1$and $\frac{r^{2}}{4\eta}<k<\frac{r^{2}}{2\eta}$，$E\pi_{m}^{RI}>E\pi_{m}^{RN}$，when $\frac{r^{2}}{2}<\eta<1$ and $\frac{r^{2}}{2\eta}<k<1$，$E\pi_{m}^{RI}<E\pi_{m}^{RN}$.

To ensure the existence of the optimal solution, the interval for information sharing is set as $\frac{r^{2}}{4}<\eta<1$ and $\frac{r^{2}}{4\eta}<k<1$

By setting $E\pi_{m}^{RI}-E\pi_{m}^{AI}=\Delta E\pi_{m}^{RI-AI}$

$\Delta\pi_{m}=\frac{1}{2}k(-\frac{(-1+\beta)(-(Q_{j}^{s}r+\gamma_{m}(L+H\rho-L\rho))^{2}-(Q_{j}^{s}r+\gamma_{m}(H-H\rho+L\rho))^{2}}{2r^{2}(-1+\beta)+4k\eta}+\frac{(Q_{j}^{s}r+\gamma_{n}(L+H\rho-L\rho))^{2}+(Q_{j}^{s}r+\gamma_{n}(H-H\rho+L\rho))^{2}}{-2r^{2}+8k\eta})$ we obtained when$1-\beta<k<1$and $0<\gamma_{m}<\gamma_{m_{1}}$and $0<Q_{j}^{s}<Q_{j_{1}}^{s}$and $\eta=\frac{r^{2}}{2}$ or$\frac{1}{2}<\beta<1$and $\frac{1}{2}<k<1$and $\eta=\frac{r^{2}}{2}$ $E\pi_{m}^{RI}>E\pi_{m}^{AI}$；when $0<\gamma_{m}<\gamma_{m_{1}}$ and $Q_{j}^{s}>Q_{j_{1}}^{s}$and $\eta=\frac{r^{2}}{2}$ or$\eta=\frac{r^{2}}{2}$and $\gamma_{m_{1}}<\gamma_{m}<\gamma_{n}$，$E\pi_{m}^{RI}<E\pi_{m}^{AI}$。

where，$\gamma_{m_{1}}=\sqrt{\frac{\gamma_{n}^{2}-k\gamma_{n}^{2}-\beta\gamma_{n}^{2}}{(-1+2k)(-1+\beta)}}$,$Q_{j_{1}}^{s}=\frac{1}{2}(\frac{(H+L)((-1-2k(-1+\beta)+\beta)\gamma_{m}+\gamma_{n}-(k+\beta)\gamma_{n})}{kr(-1+2\beta)}+L_{1}$

## Proof of Proposition A.4

The equilibrium solution considering the factor of multi-platform competition is presented as follows:

，，，$E\pi_{m}^{AI}=\frac{E[a|Y]^{2}(-4(-1+b_{o})k+r^{2}(-1+\beta))(-1+\beta)}{8(-1+b_{o}^{2})k-4r^{2}(-1+\beta)}$,$E\pi_{o_{1}}^{AN}=\frac{E[a|Y]^{2}(2(-1+b_{o})k+r^{2}(-1+\beta))\beta}{8(-1+b_{o}^{2})k-4r^{2}(-1+\beta)}$,$E\pi_{o_{2}}^{AI}=\frac{E[a|Y]^{2}(-1+b_{o})k(2(-1+b_{o}^{2})k-b_{o}^{2}r^{2}(-1+\beta))\beta}{2(-2(-1+b_{o}^{2})k+r^{2}(-1+\beta))^{2}}$，

$$E\pi_{m}^{RI}=\frac{E[a|Y]^{2}(2k(3+b_{o}(-2+\beta)-2\beta+r^{2}(-1+\beta))(-1+\beta)}{2k(b_{o}^{2}(-2+\beta)^{2}8(-1+\beta))-4r^{2}(-1+\beta)}$$

$$E\pi_{n}^{RI}=\frac{E[a|Y]^{2}k^{2}(2+b_{o}(-2+\beta))^{2}(-1+\beta)^{3}}{(b_{o}^{2}k(-2+\beta\text{）}^{2}+8k(-1+\beta)-2r^{2}(-1+\beta))^{2}}$$

$$E\pi_{o_{1}}^{RI}=\frac{E[a|Y]^{2}(k(4+b_{o}(-2+\beta)-4\beta)+r^{2}(-1+\beta))\beta(r^{2}(-1+\beta)+k(4-4\beta+b_{o}(b_{o}(-2+\beta)+\beta)))}{(b_{o}^{2}k(-2+\beta)^{2}+8k(-1+\beta)-2r^{2}(-1+\beta))^{2}}$$

$$E\pi_{o_{2}}^{RI}=\frac{E[a|Y]^{2}k^{2}(2+b_{o}(-2+\beta))^{2}(-1+\beta)^{2}\beta}{(b_{o}^{2}k(-2+\beta)^{2}+8k(-1+\beta)-2r^{2}(-1+\beta))^{2}}$$

By setting$E\pi_{o_{2}}^{AI}-E\pi_{o_{2}}^{AN}=V_{o_{2}}^{AI}$，$E\pi_{m}^{AI}-E\pi_{m}^{AN}=V_{m}^{AI}$we obtained $V_{m}^{AI}=\frac{(H-L)^{2}(-4(-1+b_{o})k+r^{2}(-1+\beta))(-1+\beta)(1-2\rho)^{2}}{32(-1+b^{2})k-16r^{2}(-1+\beta)}$，$V_{o_{2}}^{AI}=\frac{(-1+b_{o})k(H-L)^{2}(2(-1+b_{o}^{2})k+b_{o}r^{2}(-1+\beta))\beta(1-2\rho)^{2}}{8(-2(-1+b_{o}^{2})k+r^{2}(-1+\beta))^{2}}$. It is readily observed that when$0<b_{o}\leq\frac{\sqrt{2-r^{2}}}{\sqrt{2}}$and $\frac{-r^{2}+r^{2}\beta}{-2+2b_{o}^{2}}<k<1$ or $\frac{\sqrt{2-r^{2}}}{\sqrt{2}}<b_{o}<1$ and $\frac{-2+2b_{o}^{2}+r^{2}}{r^{2}}<\beta<1$ an d$\frac{-r^{2}+r^{2}\beta}{-2+2b_{o}^{2}}<k<1$, $E\pi_{o_{2}}^{AI}>E\pi_{o_{2}}^{AN}$, $E\pi_{m}^{AI}>E\pi_{m}^{AN}$

By setting $E\pi_{o_{2}}^{RI}-E\pi_{o_{2}}^{RN}=V_{o_{2}}^{RI}$,we obtained

$\begin{matrix} V_{o_{2}}^{RI}=-(((H-L)^{2}(b_{o}k(2+b_{o}(-2+\beta)-2\beta)(-2+\beta)+4k(-1+\beta)-2r^{2}(-1+\beta))\beta(12k(-1+\beta)-2r^{2}(-1+\beta) \\ +b_{o}k(-2+\beta)(-2(1+b_{o})+(2+b_{o})\beta))(1-2\rho)^{2})/(16(b_{o}^{2}k(-2+\beta)^{2}+8k(-1+\beta)-2r^{2}(-1+\beta))^{2})) \end{matrix}$. It is readily observed that when $k_{7}<k<k_{8}$,$E\pi_{o_{2}}^{RI}>E\pi_{o_{2}}^{RN}$, when $k_{8}<k<1$, $E\pi_{o_{2}}^{RI}<E\pi_{o_{2}}^{RN}$

Where $k_{7}=\frac{-2r^{2}+2r^{2}\beta}{-8+4b_{o}^{2}+8\beta-4b_{o}^{2}\beta+b_{o}^{2}\beta^{2}}$，$k_{8}=\frac{-2r^{2}+2r^{2}\beta}{-4-4b_{o}+4b_{o}^{2}+4\beta+6b_{o}\beta-4b_{o}^{2}\beta-2b_{o}\beta^{2}+b_{o}^{2}\beta^{2}}$

By setting $E\pi_{m}^{RI}-E\pi_{m}^{RN}=V_{m}^{RI}$, we obtained $V_{m}^{RI}=\frac{(H-L)^{2}(2k(3+b_{o}(-2+\beta)-2\beta+r^{2}(-1+\beta))(-1+\beta)(1-2\rho)^{2}}{8(b_{o}^{2}k(-2+\beta)^{2}+8k(-1+\beta)-2r^{2}(-1+\beta)}$. It is readily observed that when$k_{8}<k<1$, $E\pi_{m}^{RI}>E\pi_{m}^{RN}$, By setting $E\pi_{m}^{RI}-E\pi_{m}^{AI}=\Delta E\pi_{m}^{RI-AI}$

$$\begin{matrix} \Delta E\pi_{m}^{RI-AI}=(k(-1+\beta)(-b_{o}r^{2}(4+b_{o}(-4+\beta))(-1+\beta)\beta+4(-1+b_{o})k(-2+4\beta+b_{o}(-2+\beta))\beta))) \\ (H^{2}+L^{2}-2(H-L)^{2}\rho+2(H-L)^{2}\rho^{2}))/(8(b_{o}^{2}k(-2+\beta)^{2}+8k(-1+\beta)-2r^{2}(-1+\beta)) \\ (2(-1+b_{o}^{2})k-r^{2}(-1+\beta))) \end{matrix}$$

The parameter range is set as follows:

$$\rho\in[0.6,0.9],\beta\in[0.1,0.5],b\in[0.1,0.8],k\in[0.05,0.4],r\in[0.3,0.9],L=50,H=100$$

Table A.2 Simulation Results for the Brand's Equilibrium Strategy Considering Multi-Platform Competition

| $\rho$ | $\beta$ | $b$ | $k$ | $r$ | $\Delta E\pi_{m}^{RI-AI}$ | Equilibrium Mode |
| --- | --- | --- | --- | --- | --- | --- |
| 0.6 | 0.1 | 0.1 | 0.05 | 0.3 | -0.1243 | AI |
| 0.6 | 0.3 | 0.4 | 0.2 | 0.5 | 0.0867 | RI |
| 0.6 | 0.5 | 0.8 | 0.35 | 0.7 | 0.1942 | RI |
| 0.7 | 0.1 | 0.2 | 0.1 | 0.4 | -0.0811 | AI |
| 0.7 | 0.3 | 0.6 | 0.25 | 0.6 | 0.0723 | RI |
| 0.7 | 0.5 | 0.8 | 0.4 | 0.8 | 0.1554 | RI |
| 0.8 | 0.2 | 0.3 | 0.1 | 0.4 | -0.0536 | AI |
| 0.8 | 0.4 | 0.6 | 0.25 | 0.6 | 0.0987 | RI |
| 0.8 | 0.5 | 0.8 | 0.3 | 0.9 | 0.1635 | RI |
| 0.9 | 0.1 | 0.1 | 0.1 | 0.3 | -0.0975 | AI |
| 0.9 | 0.3 | 0.4 | 0.2 | 0.6 | 0.0658 | RI |
| 0.9 | 0.5 | 0.8 | 0.35 | 0.9 | 0.1579 | RI |
